# How does a (Smart) Age-Friendly Ecosystem Look in a Post-Pandemic Society?

**DOI:** 10.3390/ijerph17218276

**Published:** 2020-11-09

**Authors:** Hannah Ramsden Marston, Linda Shore, P.J. White

**Affiliations:** 1 Health & Wellbeing Strategic Research Area, School of Health, Wellbeing & Social Care, The Open University, Milton Keynes, Buckinghamshire MK7 6HH, UK; 2 Mi:Lab, Department of Design Innovation, Maynooth University, W23 F2H6 Co. Kildare, Ireland; Linda.Shore@mu.ie; 3 DesignCORE, Humanities, Institute of Technology Carlow, R93 V960 Carlow, Ireland; PJ.WHITE@ITCARLOW.IE

**Keywords:** older adults, community, aging, technology, digital, e-health, urban planning, smart ecosystem, gerontechnology, age in place, coronavirus, COVID-19, design hacking, internet of things, human-centered design, smart cities

## Abstract

COVID-19 has impacted not only the health of citizens, but also the various factors that make up our society, living environments, and ecosystems. This pandemic has shown that future living will need to be agile and flexible to adapt to the various changes in needs of societal populations. Digital technology has played an integral role during COVID-19, assisting various sectors of the community, and demonstrating that smart cities can provide opportunities to respond to many future societal challenges. In the decades ahead, the rise in aging populations will be one of these challenges, and one in which the needs and requirements between demographic cohorts will vary greatly. Although we need to create future smart age-friendly ecosystems to meet these needs, technology still does not feature in the WHO eight domains of an age-friendly city. This paper extends upon Marston and van Hoof’s ‘Smart Age-friendly Ecosystem’ (SAfE) framework, and explores how digital technology, design hacking, and research approaches can be used to understand a smart age-friendly ecosystem in a post-pandemic society. By exploring a series of case studies and using real-life scenarios from the standpoint of COVID-19, we propose the ‘Concept of Age-friendly Smart Ecologies (CASE)’ framework. We provide an insight into a myriad of contemporary multi-disciplinary research, which are capable to initiate discussions and bring various actors together with a positive impact on future planning and development of age-friendly ecosystems. The strengths and limitations of this framework are outlined, with advantages evident in the opportunity for towns, regions/counties, provinces, and states to take an agile approach and work together in adopting and implement improvements for the greater benefits of residents and citizens.

## 1. Introduction

### A Tale of Two Snows

The name Jon Snow (house of Stark) for many people will resonate with the fictional character from the television series Game of Thrones (GOT). The eighth season of GOT and the preceding seasons weave narratives featuring (dis) loyalty, treachery, war, passion, and history across various geographic locations or across the seven Kingdoms (e.g., the Kingdom of the North, the Kingdom of the Isles and the Rivers, the Kingdom of Mountain and Vale, the Kingdom of the Rock, the Kingdom of the Storm, the Kingdom of the Reach, and the Kingdom of the Dorne). Each Kingdom is represented by a ‘House’ (i.e., the Kingdom of the North) and in this example, House Stark of Winterfell—the ancestral home of Eddard (Ned) Stark; the landed gentry, including their families and the surrounding communities who are loyal to Winterfell, are depicted through the lens of fantasy and medieval history, illustrating the different cultures, expectations, loyalty, and behavior(s) of communities in different rural, urban, and city environments [1,2,3,4,5,6,7,8,9].

However, there is another John Snow, a real person who played a non-fictional part in the lives of city dwellers located in London, UK. John Snow (b.1813–d.1858) was a physician, a leader in the development of anesthesia and medical hygiene, and was most importantly known for identifying cholera as the ‘hot spot’ or outbreak in 1849 [10] in Broad Street (now known as Broadwick Street) located in an area of Soho, London.

Between 1846 and 1860, this was the third cholera epidemic, and Robert Koch, a German physician, is known to have said how cholera was “our best ally” [10] (p. 169) to improve the hygiene and sanitation of the citizens. The cholera epidemic broke out in England and Wales and lasted for two years; this resulted in 52,000 deaths, while John Snow is known as the physician to have identified a specific water pump—accessible to the public and located in Soho, as the primary source for this third cholera pandemic [10] (p. 170).

**Figure 1:**
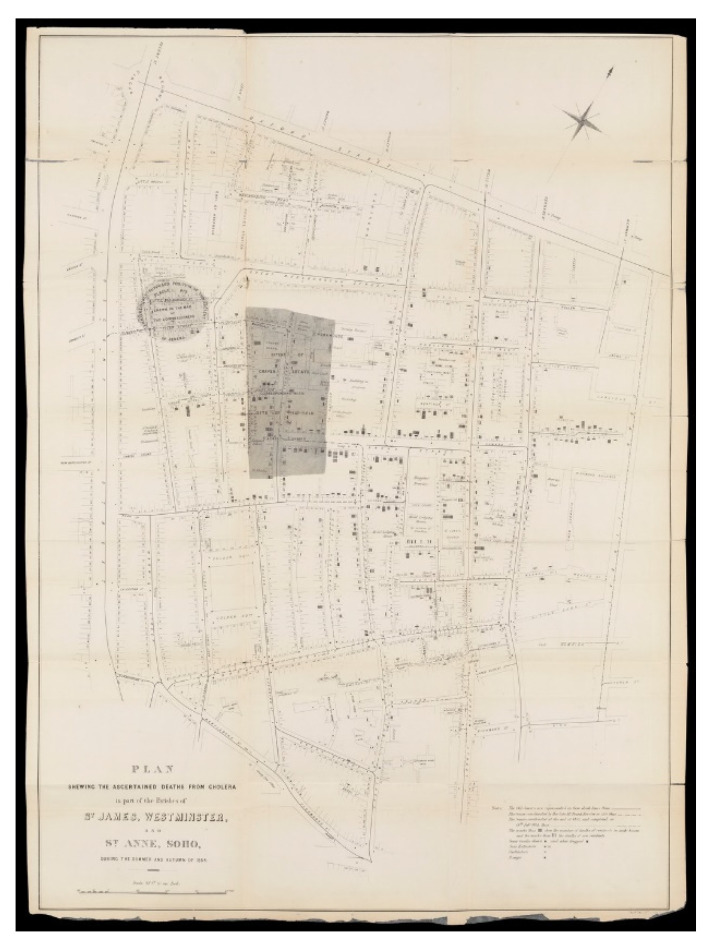
A map of Soho, London, created by John Snow to record the number of cholera deaths. Source: Wellcome Library, via Wellcome Images [13,14]. Permission granted via Creative Commons.

**Figure 2:**
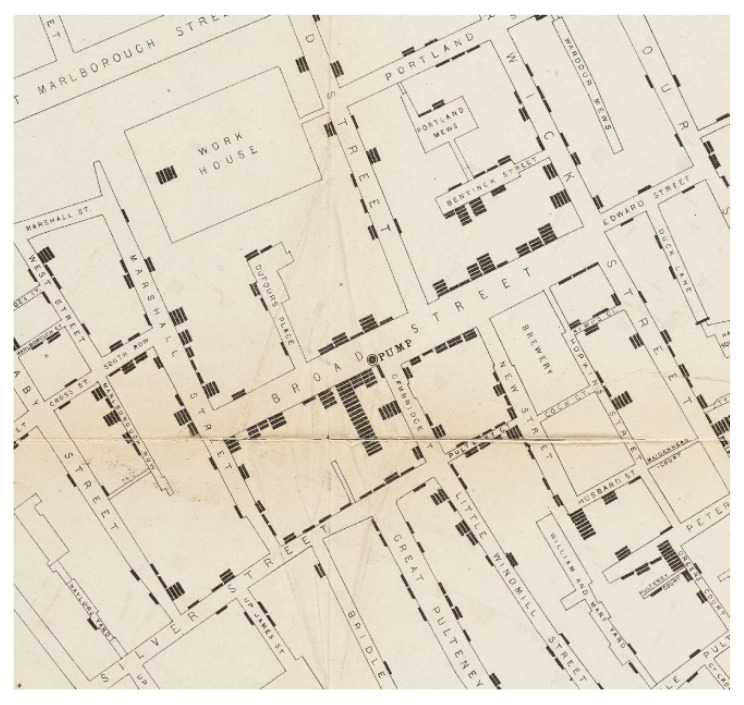
A map of Soho, London, created by John Snow to record the number of cholera deaths. Source: Wellcome Library, via Wellcome Images [13,14]. Permission granted via Creative Commons.

Although previously it was thought that the cause of this outbreak was miasma—also known as airborne particles [11,12], and as depicted in Figure 1 and Figure 2 [13,14] created by Snow, he was able to identify the source of cholera as a waterborne disease instead. Given this location and accessibility of the water pump (Figure 2), citizens in this locality who were living or visiting the area for work would drink from the pump, which in turn facilitated the disease to travel, resulting in an increase in deaths. However, some of the workmen in the area chose not to drink from the water pump but instead chose to drink alcohol from the local brewery (including men who worked at the breweries); this choice led to the bacteria found in the waterborne disease to be killed [15]. The workhouses in the area also had their own water supply, and this too resulted in fewer deaths from cholera [15,16].

However, Snow conducted an experiment using two water sources located across London in two specific areas: (1) The Southwark Vauxhall Company and (2) The Lambeth Waterworks Company, whereby water was drawn directly from the river Thames, but from different locations. The location of Site 1 was closer to the city’s sewage and Snow considered this area to be contaminated more than Site 2, which was located further up the river Thames.

Data taken from the two sites was compiled and coupled with the number of deaths in these two areas; it was noted in the location of Site 1 (Southwark Vauxhall water company) that there were 315 deaths associated to cholera per 10,000 homes, while in Site 2 (Lambeth Waterworks Company), there were only 37 deaths [17]. Having this information and data, the findings were shared and lobbied with the public health authority, which both resulted in and impacted a change to both social and sanitation across areas of London, also known as the slums. These changes were not solely conducted in London but across the country, leading to a greater improvement in health, and in turn resulting in a reduction in poor health and death of citizens [18]. This work conducted by Snow during this third cholera epidemic in the UK impacted science as well, and with the recording of deaths paved the way towards the field of epidemiology, understanding and identifying patterns [17,18].

Similarly, in the ongoing COVID-19 (SARS-CoV-2) pandemic, the first since the last global pandemic (1918–1920) that was caused by the H1N1 influenza A virus [19,20] which infected 500 million people across four successive waves. The field of epidemiology has played a key role in modelling and predicting the behavior(s) of this coronavirus [21,22], which attacks respiratory organs. Pandemics do not solely impact upon the health of citizens, but affect various “social, cultural, economic, and political,” factors [23], (p. 1), which in turn, make-up our respective society and communities. The notion of pathogen mutation was, as Ironstone notes, sounded by scientists and experts in the field of biomedicine in the latter part of the 20th century [23].

Across UK Media outlets, citizens have been informed by their respective governments that they are following the science [24,25] presented by the SAGE (Scientific Advisory Group for Emergencies) committee. SAGE includes scientists from various Russell Group Universities, public agencies (e.g., Public Health England (PHE), Food Standards Agency (FSA)), funding agencies (e.g., Wellcome Trust, and UKRI), representation from devolved governments (e.g., Welsh Government, the Northern Ireland Executive, and the Scottish Government), the National Health Service/Digital, and UK Government offices (e.g., Foreign and Commonwealth Office, Department of Education) [26]. Additionally, the Scientific Pandemic Influenza Group on Behaviours (SPI-B) also advises the Government in “anticipating and helping people adhere to interventions that are recommended by medical or epidemiological experts” [26]. The SPI-B committee have also offered advice to the Government in an attempt to deal with the various stages of the pandemic unravelling across society, at both local, national, and global perspectives [26].

Considering what Ironstone [23] notes of the impact a pandemic can have on society at various levels of the ecosystem, Jayakumar and colleagues [27] provide a series of ‘lessons learned’ from the COVID-19 pandemic. These include: (1) transformation of the healthcare section, (2) working from home is highly possible, (3) online education, (4). growth of online business, (5) need for good network communication, (6) cybersecurity is a priority, and finally (7) reconnecting with oneself and loved ones. Furthermore, this relates to the myriad of factors outlined by Ironstone [23], while also acknowledging how societal behavior has changed at present, and possibly forever.

Digital technology and social media have played an integral role in the pandemic to date across various sectors of the community. Digital technology has and is enabling (vulnerable) citizens to shop online via protected delivery slots to children receiving online education via various communication platforms (e.g., Zoom), continuing and enhancing social interaction with family members, friends, and work colleagues [27,28,29]. We have seen via (social) media posts, advertisements, and interviews, that there are many community groups (e.g., churches and choirs) and organizations have been meeting online to conduct their (leisure) activities. Many communities up and down the country organically and in a rapid response to the pandemic, have created specific community groups to provide assistance to the vulnerable people in their respective communities and areas, including those people who became ill to the virus [27,28,29].

Previously, Marston and van Hoof [30] coined the term “smart age-friendly ecosystem” (SAfE) when presenting an alternative age-friendly framework, which incorporates and acknowledges the impact that technology and physical space plays within 21st century society. As has been witnessed and experienced since March 2020, technology and its associated devices and software have and are continuing to play a critical role in the lives of citizens and society—to maintain some sense and level of normality.

The purpose, aims, and objectives of this paper are to explore how digital technology, design hacking, and research approaches can be used to understand a (smart) age-friendly ecosystem in a post-pandemic society. We provide an insight into a myriad of contemporary multi-disciplinary research, which can initiate discussions and bringing various actors (e.g., planners, scholars, health practitioners, educators, residents, developers, local, national, and international governments) together. This will, in turn, narrate future planning and development of age-friendly environments and housing in the coming decades.

The outline of this paper follows an overview of global aging in Section 2, Section 3 explores the notion of smart cities, and Section 4 discusses social isolation and loneliness followed by a series of case studies in Section 5, whereby we discuss design hacking. Section 6 explores a series of case studies from the standpoint of the COVID-19 pandemic and Section 7 proposes the ‘Concept of Age-friendly Smart Ecologies (CASE)’ framework, using real-life scenarios, and Section 8 proposes recommendations and conclusions. We aim to offer an alternative blueprint, one which incorporates technology that has been largely ignored since the conception of the first framework.

## 2. Global Aging Societies

In 2019, the United Nations (UN) reported that 16% of the global population will reach the age of 65 years and older by 2050. From a global standpoint, the UN reported in 2018 that citizens who are aged 65 years and over will outnumber children who are aged five years and under [31]. Similarly, The Organization for Economic Co-operation and Development (OECD) [32] estimate that there will be an increase of 25.1% in adults aged 65 years by 2050 across the OECD member states [32].

Furthermore, with these estimated growing projections, incurred with incredible societal shifts associated to a rise in aging populations, coupled with low birth rates, access to amenities and ensuring a community ethos that caters for all citizens across the lifespan is critical for positive age-in-place.

The relationship and engagement between aging and urbanism has been termed urban aging [33,34], in conjunction with additional attributes such as the stresses, concerns, and matters contributing to local, regional and national communities across a variety of domains of urban living [35]. Therefore, given the rise in population aging, urban change, technology innovation, societal behavior, and the need for services in conjunction with the adaptation of existing physical spaces, these factors present several challenges for many actors at local, regional, and national levels in society.

In the following sections, we provide an overview of contemporary literature surrounding the notion of smart cities and age-friendly initiatives. The work presented here is significant because it contributes to the fields of gerontology, geography, social sciences, architecture, design, social policy, design, and health by building on existing scholarly activity, policies, and is pushing the narrative of the age-friendly movement forward in an attempt to open and enhance discussions more. 

Additionally, the work presented in this paper has the potential to impact societies on both a national and international landscape, because we build on the existing and contemporary literature of the World Health Organisation (WHO) age-friendly framework [36] and the proposed extended SAfE framework posited by Marston and van Hoof [30]. The former framework was published 13 years ago, and at the time did not include technology [36], and with the recent extended SAfE framework [30], various technologies and exemplars are provided. Furthermore, given the rise in aging populations, and the differences between different cohorts (e.g., Baby Boomers, Gen X, Millennial’s, Gen Z), the barriers, challenges, needs, requirements, and expectations will vary greatly across different cohorts. The living, physical, and urban environments must be agile and flexible to adapt for the various changes that will likely be experienced now and in the future by different societal populations.

In the following section, we provide an overview and insight on contemporary literature surrounding smart cities located across different regions and countries.

## 3. Creating a Smart City for the 21st Century

### 3.1. Smart Cities

In this next section, we explore the domain of smart cities, whereby we explore and discuss contemporary literature and research.

The concept/term of smart cities has been around for over two decades [37] whereby, members of the Academy have been conducting various scholarly activity intersecting at the fields of computer science, planning, and development. Firstly, Praharaj and Han [38] provide an extensive overview of the various global debates surrounding the smart city concept, including the various terms that have previously been used and are still interchangeable in current narratives and discourses. Such terms include digital city [39], tech city [40], wired city [41], ubiquitous city [42], intelligent city or information city [43], knowledge city [44], smart community [45], creative city [46], and sustainable city [47]. For this paper, we will use the term ‘smart city’.

Smart cities [48,49,50,51,52,53,54] can offer great opportunities to reduce carbon emissions, while increasing sustainability targets, enhancing resilience, improving livability, and economic growth, coupled with the notion of increased populations [48,49,50,51,52,53,54].

Smart cities have the ability to collect and generate a vast amount of data, especially now more so than ever, given the phenomenal technological developments that have been conducted over the last 20 years. Technology innovation coupled with the notion of generating data is vast, enabling the coupling of technopoles, digital cities, and intelligent cities, affords various actors, organizations, and governments greater opportunities to exploit the generated data [50]. This, in turn, offer urban planners and other key actors the opportunity to respond to the myriad of societal challenges posed in the modern 21st century society.

Allam [48] provides an overview of the various concepts and differences surrounding digital, smart, and intelligent cities. The concept of a digital city is compounded by the integration of digital technology into the mainframe of the cities’ infrastructure and systems, in conjunction with transport and buildings, which are seen as areas of the societal ‘fabric’ woven and embroidered to facilitate the process by which the data can be collected and processed.

However, the concept of a ‘knowledge city’ by Edvardsson, Yigitcanlar, and Pancholi [50] is associated with a greater breadth of dissemination and knowledge translation within the segments of the ‘urban fabric’, including both the public and private sectors of the societal ecosystem. This notion at the time of conception (1990s) relied on what is now referred to as ‘big data’; yet, in the 1990s, this type of data was not available [53]. Since the 1990s, the term and concept of ‘technopoles’, coined by Allen J. Scott [53,54], relates to regions that are primarily focused on technological innovation [53,54], comprising of different components such as local business, educational institutes (e.g., universities), financial institutions, and public research organizations. Such developments are usually created either by the: (1) private sector, or (2) in partnership with both public and private sectors [55]. Moreover, the ‘smart city’ concept varies differently from the other concepts because it relies on the intelligence of digital devices and deployment across the physical space to improve the ‘urban fabric’, the economy, and the lives of the citizens [56,57,58,59].

The smart city [60,61] concept, in conjunction with the deployment of sensors, devices, and cameras that are interwoven across the infrastructure and mainframe of a city, has the potential and capability to generate various forms of data [60,61]. There are several examples of how the Internet of Things (IoT) can and are being integrated into real-world environments, such as the home, which include security systems—doorbells, smart heating, and lighting devices. Previously, Marston and van Hoof [30] described several IoT devices in their respective paper, describing various scenarios illustrated by existing real-life examples. Additionally smart/virtual devices assistants such as the Amazon Alexa or the Google Mini [62] can provide further information to citizens in the home environment, including medication reminders, calendar reminders, and leisure activities (e.g., selecting and listening to music) [62,63].

More recent application of smart devices and IoTs are starting to be integrated into kitchen appliances, such as washing machines, dishwashers, and coffee machines [64], enabling automatic reordering of washing machine powder/tablets, dishwasher tablets, and coffee beans when stocks are running low. Furthermore, smart cities provide regional, national municipalities/governments, citizens, and residents the opportunity to meet challenges surrounding carbon emissions, energy consumption, and traffic infrastructure [61]. While these examples are primarily focusing on the smart home environment, and in the context of smart cities, real-life examples of IoTs include reservation of car parking spaces or tracking usage of bicycles across a city (e.g., Copenhagen, Denmark) [62]. Coupled with the growing body of literature and research, which is exploring how cycling can be utilized in the context of big data, IoT, monitoring fleets, connected programs proposes programs such as the ‘Smart Velomobility’, which explores and considers both political and practical approaches to smart cycling [57,58,59,61,65,66,67,68].

Moreover, IoTs and associated technologies can be implemented into the context of ‘Innovative Smart Grid Technologies’ [57,58,59,61,65,66,67,68], which facilitates sensors and components to be integrated into the infrastructure of a city to track the energy consumption of residents [69]. While automation can play a role in detecting and changing power consumption, blackouts, and fuel loads, which, in turn, facilitate safety and economic savings to residents [69]. In towns such as Milton Keynes (along with other towns and cities), there are charging stations for drivers of electronic vehicles (EV), which enable EV owners/drivers the opportunity to ‘recharge’ their EV as well as continue to use their respective smart technologies should power outages or accidents occur [69].

Shin [70] provides a detailed understanding of the IoT ecosystem in the context of Korea, comprising of a multi-level analysis, which incorporates users, the wider society, and ecology. Findings from this in-depth study, which implemented the social construction of technology (SCOT) approach as a means of understanding how useful IoTs could be within a multi-level society, was studied. However, employing a socio-technical theory facilitated the intervention of appropriation as a social construction within society, which is interwoven and intersects at the differing levels within a respective ecosystem [70]. To date, Shin notes how there has been “too much emphasis on technology in IoT project” (p. 92) and by taking the respective study as a means of unpacking this statement, Shin highlights how there are many obstacles within projects, which lean more towards the social rather than the technical, and this includes privacy, universal service, and the digital divide. Shin purports that the technological lag of Korean development may not be related to the availability of technology but instead could be due to the lack of user/resident demand, which in turn transfers to a greater price increases for products [70].

Conversely, Shin [70] describes several negative concerns identified from the study, which includes the perceived understanding of IoTs from a top-down approach by the Government and the impact this may have on citizens. However, citizen concerns were noted and include the security and privacy concerns of the information exchanged from within the confines of a safe and secure socio-technical ecosystem, resulting from a potentially less secure external ecosystem and network(s). Additional concerns were highlighted by citizens in the respective study relating to existing wearable and IoT devices executing covert behavior or harvesting and sharing data unbeknown to the citizens/user(s) [70]. This concern leads to the ethical considerations of using, and implementing such devices not only on an individual level, but across wider spectrums of the ecosystem; Shin states that “without adequate and timely policies regarding these matters […] smart cities cannot be successfully developed in future”(p. 92) [70,71,72].

Seven recommendation points are proposed by Shin [70] to move the narrative forward and gain a congruence associated to both ethical and socio-technical frameworks. The recommendations include: 1. Consider people before local context; consider local context before technology, 2. Demonstrate sustainability, scalability, and resilience over a long-term timeframe, 3. IoT of any new development should conform to the best available current standards for interoperability between IT systems in general, 4. New developments should demonstrate that they have considered the commercial viability of providing digital civic infrastructure services, 5. The government should support a meaningful IoT literacy program and raise awareness to empower self-regulation and enhance individuals’ interactions with IoT, 6. Social demands should not be identified and addressed solely by the market. The boundary between the social and the economic must blur, and society must be reshaped into a more participatory arena, and 7. Users must be empowered to utilize IoT technologies to turn the physical environment into a socio-technical environment, where appropriate policies are shaped around them.

Concluding from his study, Shin [70] states that “Deployment of IoT is not far from reality. Unlike previous smart city initiatives, IoT will, and should, exist for a long time. For the time being, however, IoT remains more of an untested promise than a reality.” (p. 97). Furthermore, and based on the findings from the respective study, and existing literature [71,72], Shin notes that there is still a lack of understanding of the positive and prospective benefits that IoTs can bring to society and ecosystems alike; and suggests Korea “may need a series of socio-technical experiments that emphasize both the sociological and technological aspects of development” [70] (p. 98). Emphasizing further, Shin describes how the infrastructure of an ecosystem should be perceived as an ‘artifact’ and taking an ethnographic approach is appropriate when designing and developing IoT sensors and devices. This though in turn will afford interested parties from industry, government, stakeholders, researchers, and designers the opportunity to identify and understand the impact of IoT via different user narratives [70].

Taking into account the proposed recommendations by Shin [70] local, regional and national governments, stakeholders, and actors have the opportunity to purvey big data which can in turn offer insight into and provide guidance to urban planners; for example, when expanding the smart city infrastructure into different locations [71,72,73,74]. Furthermore, Allam [75], Allam and Dhunny [76] notes such a concept of a smart city offers various actors the opportunity to collaborate, and utilize the various data from across the infrastructure to reach optimal usage, including the maximization of resources and technologies available within the infrastructure, buildings, and the urban fabric. Potentially, this in turn offers sustainability, feeding into outlets of the ecosystem.

In today’s society, we have seen the potential possibilities of integrating technology and IoTs IoT into various urban developments, towns, and cities, as described by Marston and van Hoof [30], who posited the notion and integration of technology into the urban environment and physical space of Milton Keynes (MK). The MK: Smart project [77] (2014–2016) aiming to focus on the new town Milton Keynes, located in the county of Buckinghamshire, UK, was a consortium comprising of various partners, including Milton Keynes Council, Anglia Water, British Telecom (BT), consultancy partners, civil engineering, charities (e.g., Community action: MK), academic partners (i.e., University of Bedfordshire, University of Cambridge), and Catapult hubs. The concept of the MK: Smart project was to bring together various actors intersecting across the areas of education, business and community engagement, and service providers.

Given the growing technological developments, this notion is conceivable based on the existing IoTs/digital devices/sensors, and potential AI capabilities, which in turn offers compliance with sustainability targets and data generation [70,71,72,73,74,75,76,77]. Platforms facilitating IoTs and machine learning offer the interwoven sensors the ability to be connected [70,71,72,73,74,75,76,77], resulting in data transfer across the mainframe of a city. Allam and Dhunny [78] note how cloud computing affords data storage from the data generated across a smart city; while blockchain technology offers data security during transfer between nodes installed on the mainframe [79,80,81,82,83]. Data privacy is key in all aspects of society, and a smart city is no different; therefore, it is key to ensure the data that is generated within the infrastructure and mainframe of a smart city, during the different processes or generation, transfer and usage, is secure. This is imperative in ensuring for actors, residents, and citizens alike that the privacy of data is maintained, and no data breaches occur [80,81,82,83].

Obedait and colleagues [84] posits the concept of a smart city in the UAE and provide an overview of contemporary literature surrounding technology implementation within the smart city concept, development, and citizen centric governance. The former concept explores the various technologies that can be integrated into such developments, including:

- Internet of Things (IoT): which facilitates the interconnections of physical devices (e.g., sensors) with buildings and other digital devices, which in turn affords data to be exchanged.
- Augmented Reality (AR) and Virtual Reality (VR): offers specific businesses such as retail and real estate according to Obediat et al. [84]. While Alkhamisi and Monowar [85] posit how AR and VR have the potential to impact and redefine the governance of business.
- Big data: refers to the large data collected through various technologies and devices. The processing of big data has the potential to provide predictive insight into user behavior analytics, which in turn can provide information relating to service provisions—health, crime/policing, and business. This in turn has the potential to impact local, regional, and national policies, agendas, and governance [86].
- Blockchain: utilizes cryptography (e.g., Bitcoin) to ensure that the verification and storage of data is safe and ensures security is maintained at all levels. Transactions between governments, regional councils, service providers, and citizens could be streamlined via the implementation and use of Blockchain.
- Artificial Intelligence (AI): utilizes machine learning techniques [87] and has the potential to refine the customer experience via local, regional, national governments, and council agencies.

The scholarly work by Obedait and colleagues [84] relates to the concept of a smart city in the UAE, as a “pioneer and leader in providing best in class citizen services utilizing technology” (p. 78). Such a location as the UAE, which includes many citizens from residents to tourists, non-residents, expatriates, and workers, needs to ensure all needs are met. Many residents and workers are younger and mobile, having arrived in the UAE for employment purposes and reasons [84] coupled with the diversity of citizens. Obedai and colleagues [84] note how this variance within their society adds additional complexities to service provision of governance for all citizens, not solely one category of citizen. Therefore, the government of the UAE has rolled out an e-government portal in Abu Dhabi, enabling citizens, tourists, and businesses the opportunity to access government services in a cost-effective approach, which also enables answers to be provided to questions and complaints can be shared [84].

Within this portal [88], there are several elements that have been implemented and include: (1) UAE national identity card, (2) happiness meter, (3) Electronic Land Management System, (4) smart district guidelines (e.g., for developers expanding the across the city), (5) smart Dubai index (gauges impact relating to the implemented initiatives), (6) Dubai data (all key information collected will be shared with citizens and government(s) moving towards a participatory government), (7) Smart Dubai Platform—which relates to the integration of IoTs across the city infrastructure, and captures data in real time, and citizens are notified via dashboards, and (8) the Dubai Blockchain, which will offer secure and improved data transactions across the city, between the various service providers, government, citizens, and tourists.

The UAE 2021 vision [89] and the UAE Government strategy [90] have provided their vision and roadmap to transform the UAE into a smart ecosystem, which encompasses a livable and resilient city that aims to be achieved by improving the connectedness of the city. This vision aims to connect citizens in Dubai via the various services, as well as enhancing the quality of life via technology, which may enable greater streamlining of different societal aspects (e.g., social, cultural, education, and healthcare) [88,89].

In summary of this section, we have provided a contemporary overview of research and insight into scholarly activity surrounding smart cities and associated technologies that have been implemented and trialed across both Western and Eastern societies. In the next section, we discuss the contemporary literature surrounding the age-friendly initiatives.

### 3.2. Age-Friendly Initiatives

Contemporary literature surrounding the age-friendly initiative has previously been discussed at length by Marston and van Hoof [30]. However, one review by Lim, Edelenbos, and Gianoli [90] aimed to explore the development of a smart city, and this piece of research reviewed 55 papers, comprising of 12 positive and four negative results.

The positive results highlighted six papers primarily relating to theoretical concepts, which did not include evidence, while the other six papers included six themes: (1) enhancing citizen involvement, (2) protecting environment, (3) facilitating social development, (4) facilitating sustainable development, (5) fostering innovation, and (6) increasing social capital. Regarding the negative results, two of the four papers were categorized as theoretical in relation to privacy and security issues, and secondly, the notion of moderating freedom of speech and democracy.

Additional reviews and research within this domain include the work conducted by Cocchia [91], who explored the concepts of a smart city and of a digital city between 1993 and 2012. The Anthopoulos and colleagues [92] review highlighted seven applied domains in relation to smart cities: (1) resource, (2) transportation, (3) urban infrastructures, (4) living, (5) government, (6) economy, and (7) coherency, describing in some instances the notion and relationship between smart cities and sustainability. Furthermore, Traindade and colleagues [93] conducted work within the area of sustainability and smart cities, and more recently Komninos and Mora [94,95] reviewed the literature between 1992 and 2012, which purported and described the development of a smart city.

Ruza and colleagues [96] conducted research in the area relating to the age-friendly factor, specifically focusing on the Palo Alto area of California; this in turn resulted in the development of a framework, encompassing several criterion and assessments deployed by a web-based geographical information system (GIS).

The rationale by Ruza and colleagues [96] for choosing the Palo Alto area was based on the regional population characteristics (i.e., high income, greater proportion of older adults in comparison to the USA overall), and providing the opportunity for this particular population to continue living in this community. One further reason for this choice of research area was the ease of access for the research team.

Findings from this research highlighted three key elements that should be taken into consideration when aiming to improve the respective community and physical space: (1) open spaces, (2) public transportation, and (3) services for an aging population/community [96]. Furthermore, Ruza and colleagues [96] note how their results align with findings from the ‘Community Services Department of the City of Palo Alto’ [97]. Coupled with the additional changes to this region of California, they have the potential to reduce the existing marginalization of community members from lower socioeconomic status [96]. From the standpoint of enhancing public transport in this region, members of the Palo Alto region own at least one car per household. However, the findings from this respective study note the need for greater improvement and accessibility of public transportation services based on prospective health and wellbeing issues in later life [96]. Ruza et al. [96] acknowledge that this region of California is an urbanized area, and previous planning developments were conducted without the considerations and issues surrounding an aging population, and with this mind, purport the following:

“[…] urge planners and decision makers to act on the items high-lighted in this study, as a lack of action will translate to escalating unmet needs and make Palo Alto unsustainable with respect to its healthcare resources and provision for its residents to age in place”

(p. 395) [96].

**Figure 3:**
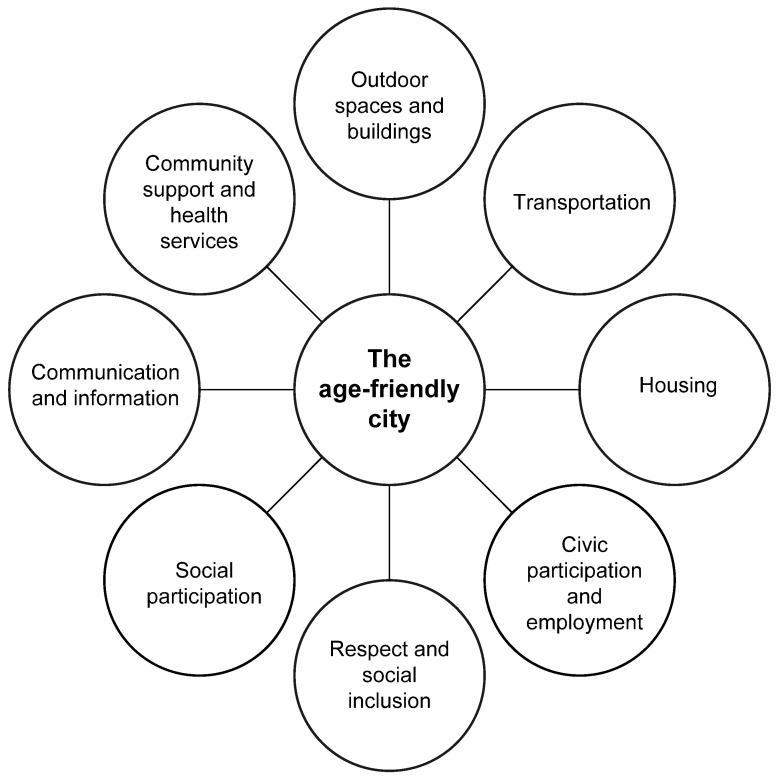
The eight domains of an age-friendly city [36].

The scholarly activity by Meijer and Bolivar [98] focused on the concept of smart urban governance and concluded this notion was between the collaboration of citizens and technology. The Centre for Ageing Better [99] based in the UK highlights the number of towns and cities that have received age-friendly status (n = 40) via support and engagement from stakeholder organizations, residents, and the leadership of respective towns and cities. Taking the lead from the eight domains (Figure 3) published by the WHO age-friendly framework [36], 1. Outdoor spaces and buildings, 2. Transportation, 3. Housing, 4. Social participation, 5. Respect and social inclusion, 6. Civic participation and employment, 7. Communication and information, and 8. Community and health services (Figure 3), the Centre for Ageing Better state, “Together, the eight domains and programme cycle create the framework for how places become increasingly age-friendly” [36].

Therefore, given how the notion, narrative, and discourse surrounding the age-friendly movement has continued for over a decade, utilizing the existing eight domains as a blue print, there is still little discourse to providing alternative, extended frameworks to the existing WHO age-friendly [36] with the exception of the work proposed by Marston and van Hoof [30].

**Figure 4:**
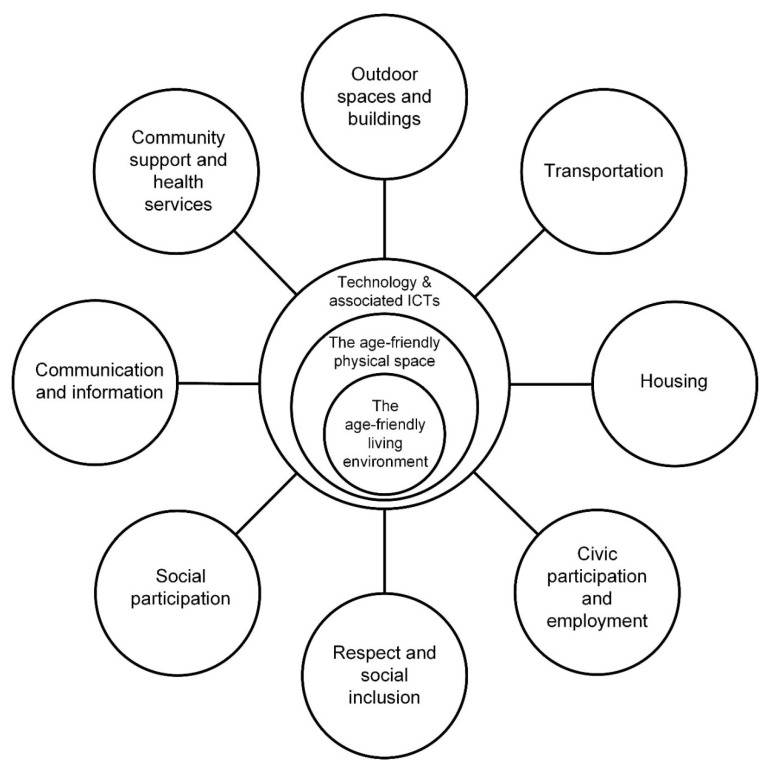
Smart age-friendly ecosystem framework (SAfE) [30].

Figure 4 illustrates the extended proposed framework by Marston and van Hoof [30], which at the time did not drill down into the different types of technology and peripheral solutions that could or should be considered in future smart age-friendly ecosystems. However, technologies and their associated software solutions (e.g., big data, Blockchain) do have a place in the design and revamping of existing and future proposed age-friendly ecosystems. It is also suggested by the authors that the adoption of new and smart technologies should consider age, gender, and personality traits [100], taking into consideration standpoints from both contemporary and post-pandemic societies. Therefore, such frameworks would differ and interact greatly or not when citizens globally have had their lives and society turned upside down during this pandemic.

In 2007, the age-friendly framework [36] was published by the WHO, and at this time, the Internet was accessible, videogame technology (hardware and software) was being used and tested for rehabilitation purposes, as well as exploring its use and medium with non-traditional audiences (e.g., older adults) in regard to cognition [101,102] and fun, [103,104,105,106,107,108]. Mobile technologies were developing at a phenomenal rate, whereby now we see the use of smartphones alongside mobile apps (mApps) and mobile health apps (mHealth Apps) [109], which are accessed and used by many citizens in their own respective ecosystems for a myriad of reasons [110].

This in turn has resulted in the field of gamification [111] and while Deterding and colleagues [111] discuss gamification from the standpoint of videogames and design, it has been part of our society through the activity of reward points (e.g., groceries, air miles, or petrol consumption) for many years. Much of the scholarly activity was published after the WHO age-friendly framework [36] was published, although at the time of designing this framework, there should have been some acknowledgement and/or theoretical discussion posited to the future and potential impact and role(s) played by technology within society.

Moreover, one of the areas that was not discussed by Marston and van Hoof [30] or by the WHO [36] was privacy and the surrounding issues associated to citizen’s data and confidentiality [112,113,114,115]. In the first decade of the 21st Century, technology developments were witnessed and experienced in society (e.g., social media platforms, videogames, mobile, and smartphones) [116,117], including research and development of mobile apps (mApps), mHealth Apps [104], virtual assistants (VA) [62,117,118,119,120], and robots [121,122]. These technologies hold a user’s data and thus privacy should also be taken into consideration when a technology is implemented.

The concept of a living lab (LL) is not new, and LLs allow multiple actors to collaborate with regard to design, development, testing, and evaluation phases to reach the goal(s) of innovation situated within a real-life environment [123,124]. The LL approach ensures full inclusivity of users who are driving the innovation, across the different phases, taking a co-creation approach aimed at services, products, and/or societal infrastructures such as smart cities [123].

Indeed, the work by Shin and Park [124] presents the concept of LL as an approach to understand the implementation of IoTs across three levels: 1. *Macro LL:* Constellation (ecosystem surrounding IoT), 2. *Meso LL:* innovation project and 3. *Micro LL:* user experience and acceptance. Ng and Wakenshaw [125] note how design and development is based on the needs of the user(s), established around a human-centered approach and system [70,126]. Moreover, the findings of the work by Shin and Park [124] who describe Bukchon Village in Korea as a real-life LL (the municipality implemented a top-down approach and did not initially consider user participation at its core) may be problematic for technology appropriation. Furthermore, Shin [70] notes how IoT implementation into a LL ecosystem can have several challenges, while taking a socio-technical approach does afford user participation from the conception stage. This allows users, stakeholders, industry, business, policy makers, and government officials to contribute across the three levels of a LL, while learning and understanding the different and meaningful experiences within this ecosystem. Lidtke and colleagues [127] note how the use and implementation of LLs have not been evaluated by academics, to ascertain whether this is a suitable approach to understanding the use and acceptance of technology and IoTs while implementing a co-design approach [128,129,130,131].

At the beginning of the pandemic outbreak and as the months continue, technology and communication tools have become integral features, playing a significant role in what the authors of this paper are coining the ‘Concept of Age-friendly Smart Ecologies (CASE)’ framework.

For many digital technologies and citizens, digital technologies have become an integral component within various ecosystems, as a means of continuing social engagement, reducing loneliness and isolation, in addition to maintaining and delivering a level of stability in education, support, and employment.

Finally, a recent review by Torku and colleagues [132] identified 81 publications and selected 39 papers for inclusion based on criterion relating to the “barriers that hinder the implementation of age-friendly initiatives in smart cities” (p. 1). Findings from the review identified several facets associated with 1. physical, 2. environmental, 3. technological, 4. social, 5. Financial, and 6. political barriers that smart cities currently experience or may experience by employing initiatives from the age-friendly cities narratives. The respective authors provide implications of this work for policymakers by suggesting that this review “would support policy makers in formulating policy recommendations to improve age-friendliness in cities” [132] (p. 1). Finally, Torku and colleagues [132] detail that the work presented in this systematic review compounds a myriad of features that may provide existing age-friendly initiatives into smart cities.

In summary, this section has explored and discussed contemporary research surrounding different age-friendly initiatives, frameworks, and research, highlighting that there is no one-size-fits-all model. We have also described various technologies that have become integral within contemporary society and offered an insight into how taking a user-centered approach to understanding technology through LLs can be a positive way of agile research, understanding how a piece of technology may or may not work within different settings.

In the following section, we explore the notion of social isolation and loneliness, which can affect both young and older adults, at various times of one’s life.

## 4. Social Isolation and Loneliness

Loneliness and social isolation [133,134,135,136,137,138,139] have been known to be key contributors to one’s poor quality of life and life expectancy and can be serious for older citizens with pre-existing chronic health conditions [140]. Scholars [139,140] note how the impact of chronic social isolation and loneliness are related to one’s poor physical and mental health, while the experience of temporary or transient social isolation and loneliness posits fewer risks relating to the long-term negative impact on one’s health and wellbeing.

Social isolation and loneliness can affect anyone across different age cohorts and throughout the life course. Be it a young person who has moved to a new region or country for employment or education purposes or older citizens who have continued to live in their home/ area, yet members of their family such as children or grandchildren have moved away, or the older person experiences a sense of loss through bereavement.

Dinkins [141] notes how socially engaging and interacting with friends, family members, and the wider community ecosystem forms part of being a human and is underpinned through positive health and wellbeing. Therefore, it should be ensured that interaction and social engagement can be accessed, in an attempt to reduce loneliness and social isolation, which in turn improves and/or enhances the overall health and wellbeing of citizens in society (including friends and family).

Contemporary research [142,143] demonstrates the importance and role that technology can play in reducing loneliness and social isolation. Schlomann and colleagues [142] ascertained positive findings from their quantitative study aimed at understanding ICT use by adults aged 80+ years. Adults in this study used ICTs in conjunction with their daily activities and reported a decrease in loneliness. However, Schlomann et al. [142] concluded how there is still an age-related digital divide and proposed the recommendation of further ICT training to reduce this difference.

Cotton and colleagues [144] conducted a study to understand the impact of Internet use on loneliness and perceived social isolation. This study was conducted in two living environments: 1. assisted living and 2. independent housing environments. Findings by Cotton and colleagues [144] ascertained participants who were recruited from one of the two living environments located in Alabama, USA, showing how technology can facilitate older adults to stay connected and meet new people. The amount of communication with other people increased and offered comfort to the older adults who experienced greater connection with friends and family. This in turn, resulted in an increase in quality of communication, and feeling less isolated.

Another study using the ‘Personal Reminder Information and Social Management (PRISM)’ system was conducted by Czaja, Boot, Charness, Rogers, and Sharit [145]. This multi-site randomized field trial was conducted across three locations and recruited 300 people who were living independently and were identified at been at risk from social isolation. Findings showed, over the course of six months, that the sense of loneliness was reduced significantly, and an increase in perception of social support and wellbeing was noted. Czaja and colleagues reported that for participants who were using the PRISM system, the findings showed improvements from baseline and at 12 months, with further increases and improvements in computer self-efficacy, proficiency, and comfort of using computers at both six- and 12-months phases. Similarly, these findings support the growing body of evidence as presented by Cotton and colleagues [144], Schlomann, and colleagues [142]; whereby the notion and access to technology and its associated applications have the potential to support social connectedness and reduce loneliness and social isolation.

Conversely, there is the need to start exploring and understanding the challenges and issues surrounding future aging cohorts (for instance, Generation X, Millennials, and Generation Z) [108,146,147,148,149,150,151]. This is important because, for various cohorts who have grown up in a digital society, with differing mental models [62] to those of existing older populations, it is important to understand the various mental models employed by different users during technology adoption as well as in earlier stages, such as the design process [62].

Citizens build and expand their mental models through various lens and experiences, which in turn impact their perception of adopting new technology into their lives and individual ecosystems. For example, citizens categorized as Generation X will still be able to remember what society was like prior to the use and integration of the Internet, and mobile/smartphone devices. However, Millennials and Generation Z do not have this previous experience and therefore, for these younger cohorts, it could be very difficult to comprehend not accessing or using such technologies. Therefore, while starting to understand the behavior, needs, and challenges that currently face younger citizens in society, there is the opportunity to plan and react efficiently to these future aging populations who may or may not have different expectations to our existing aging populations (e.g., Baby Boomers, Oldest Old) [147,148,149,150,151].

In summary, this section has presented research surrounding the use of technology by older adults in a bid to alleviate loneliness and social isolation. Although respective studies reported positive findings, technology is not a quick fix solution or replacement for face-to-face interaction. Technology should be perceived as an accompaniment to the user and/or older person, as a means of adding another layer of connectivity. Furthermore, we discuss how Generation X should be considered in future research to understand the appropriation of technology and user experiences from the standpoint of a different cohort, who has different experiences to Baby Boomers, Millennials, and Generation Z. Presently, Generation X has little attention from scholars across the fields of gerontology, gerontechnology, and human computer interaction (HCI). However, given the intersection of these fields within this multi-and-inter-disciplinary domain, we believe it is important to commence exploration of what the exact needs, challenges, and barriers are faced from Generation X [148,149,150,151].

In the following section we discuss a myriad of design hacks, which can afford citizens, businesses, academe, policy makers, and stakeholders the opportunity to retrieve rich information that in turn can be implemented into practice.

## 5. Design Hacks 2020

Societal and environmental sustainability relies on design approaches [152] and a universal design [152,153,154,155] approach is recommended when designing for older adults. This approach has been applied previously as a means of gaining user insights early on in the process [153] and highlights the importance of taking three facets into consideration: 1. design requirements, 2. user comfort, and 3. ease into account. Likewise, the challenges and experiences of aging can be understood by the implementation of this design approach, whereby this approach aims to solve and address unmet needs [156,157]. Furthermore, design fiction enables us to speculate and critique current and future scenarios about how things could be [158]. Employing universal design approaches and participatory design tasks enables designers, researchers, and users the opportunity to be innovative, while understanding unmet needs, barriers, and challenges, which may or may not be experienced. Furthermore, these approaches also afford the research team understanding, which highlights the positives and benefits of innovative technology design, while becoming less reliant on ‘self-reporting’ of environment experience (e.g., walkable neighborhoods or social drift) [158,159]. This collaborative approach draws on existing universal design principles between design teams and various age-friendly stakeholders, including older adults who have the potential to collaborate in a co-creation process successful and satisfying products, systems, and environments [145,156,157,158,159].

### 5.1. Case Studies

Design hacking is all around us; it is how we can personalize or adapt technologies or products to offer greater experiences or enhance our existing understanding and perceptions. This, in turn has the potential to increase and enhance our health, wellbeing, and quality of life. In contemporary fieldwork and research activities, design hacking has become an integral component of enquiry, and can give multi-and-inter-disciplinary teams the opportunity to facilitate this approach as a way of understanding appropriate pathways and routes to revaluating and reframing age-friendly environments and experiences [158,159].

The following case studies, which share the experiences and narratives of four older adults, describe and illustrate how they have overcome product challenges by ‘hacking, adapting or preparing’. Names have been changed to maintain agreed anonymity between the participant and the researcher.

#### 5.1.1. Case Study A—Mary (2016)

Mary lives alone and has been separated from her spouse for several years; she has two adult children. Mary still works as an art teacher/therapist on a part-time basis. Her front room is a studio, which is full of materials, paints, and books. Mary has macular degeneration and arthritis and takes daily medication for various health conditions. During one of the fieldwork sessions, the following information was revealed: *Marys’ arthritis medication has consistently managed her condition well and she has not experienced any challenges or difficulty, until she received her last prescription from the pharmacy. Although the medication was not changed by the pharmaceutical manufacturer, the manufacturer did decide to ‘update’ the packaging which includes a blister pack which holds each of the pills. The material of the blister pack is made of a harder, tougher type of material than previous packaging. The font on the packaging has also been changed and is presented diagonally rather than horizontally (left to right) as previously printed. Additional changes have also been made to the style of the font, which is now presented in a less bold and readable font style. This in turn has led Mary to experience difficulty reading the information as she expresses, “If I didn’t know this was my medication, I wouldn’t be able to read it”.*

These small changes by the manufacturer have led to greater difficulties for patients such as Mary, which in turn has led to greater difficulties and confusion because of her health condition—macular degeneration. Furthermore, Mary is experiencing greater issues in relearning or recalibrating her mental models to recognize the new pill packaging alongside her other existing medications. Additionally, due to the changes in the physical packaging of the medication, Mary expresses how she experiences increased pain from the pressure of trying to open/access the tablet through the blister pack.

The solution to ease both discomfort when accessing the tablets is that Mary’s daughter will remove each of the tablets (1-month supply) from the packaging and store them in a jam-jar, which in turn enables Mary to easily access her medication. Mary has tried another option to access her medication, which includes using a spoon to burst or ‘pop’ open the blister packaging.

#### 5.1.2. Case Study B—Joan (2014)

Joan’s home has a colorful entrance with flowers blooming on each side of the pathway leading to her entrance door. Joan is an avid gardener and loves any opportunity to work outdoors making her garden look beautiful. Joan is widowed and lives alone. Her adult children and grandchildren visit regularly, and the family ties are close.

**Figure 5:**
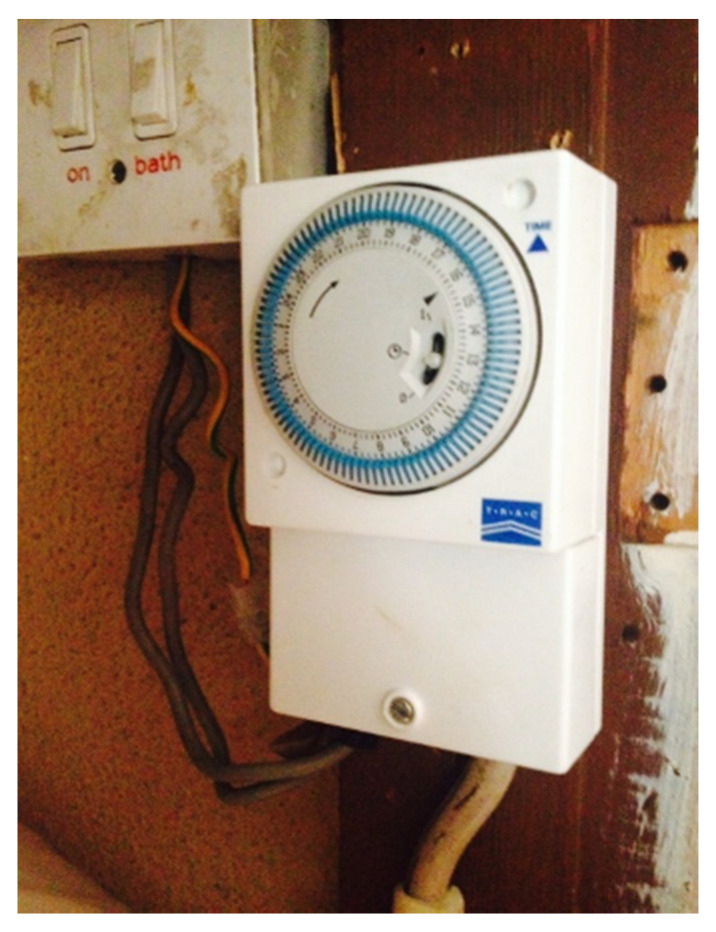
The interior of Joan’s ‘hot-press’, displaying the central heating timer and immersion heater on the left, and torch on the right. Permission granted by Dr. L Shore.

**Figure 6:**
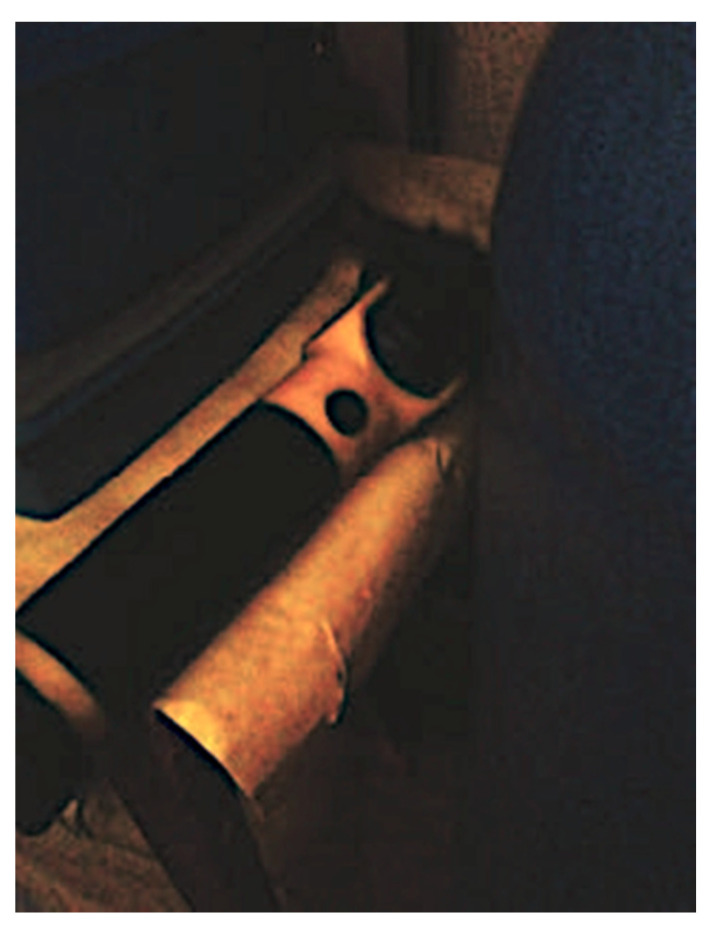
The interior of Joan’s ‘hot-press’, displaying the central heating timer and immersion heater on the left, and torch on the right. Permission granted by Dr. L Shore.

The maintenance and access of various systems in the home were discussed. Joan highlighted how her central heating timer and immersion switch to heat her water are in her ‘hot-press’ (Figure 5), which is the key source within her home for hot water supply. This hot press has no light, and the dials can be difficult to read and see. As a solution, Joan keeps a torch in the ‘hot-press’ (Figure 6), which affords her to see and read the visual setting of the central heating timer and immersion more easily. As an additional back-up as well as forming part of the consideration of this important aspect of comfort and hygiene, Joan keeps a supply of batteries to ensure the torch consistently works. This planning and consideration to tasks and home management is an example of challenge and opportunity to retro fit and update older homes with newer technologies that can offer improved ease of access.

#### 5.1.3. Case Study C—William (2014)

William lives in a cottage with his wife in a rural area of Ireland. Several years ago, the cottage was extended with extra rooms and a new entrance doorway. This construction was started pre-retirement and because the planning commenced alongside active discussions regarding the type of home improvements, workflow and accessibility was required in their home.

At the beginning of this construction project, William and his wife considered their future selves and how the new rooms and access would impact their mobility, while also considering how they could implement an appropriate physical space to benefit and support them in later life, through their aging experience.

During this process, they factored in the idea that at some stage, either William or his wife could lose or encounter mobility challenges. This thought led them to consider the ‘what if’ question(s), relating to ‘what if they were spending time in a wheelchair’ or ‘what if I was using a walking stick’, or how easy would it be to live at ‘home’.

With these types of questions in mind, they considered one significant feature, which they introduced into the physical space outside of their home, and this was to ensure that they had access to footpaths surrounding their home. This was important also in case they needed a level or ramp built to assist either of them, should they be in a wheelchair. This type of thought and consideration helps in maneuvering the device independently or ease the strain of pushing a wheelchair should a family member or friend be helping either of them onto the pathway.

Additionally, they were also intending to offset the potential ‘disruption’ of ‘reactive home updates/renovations after an event has occurred, by actioning this foresight—the familiar pathway features would age with the building and not appear as a reminder to the experience of reduction in mobility.

These thoughts, considerations, and expressions were important conversations between William and his wife, which he relayed to the researcher. Furthermore, this thought process, and planning for the future, endorsed how as we age in place [159], acknowledging the physical and mobile aspects of aging, and can be prevented by exploring alternative considerations to enhance comfort within the individual home ecosystem. Furthermore, conducting physical changes to the home prior to retirement will ensure less disruption and construction works in the future when life and respective situations are more sensitive.

#### 5.1.4. Case Study D—Jane and Remote Sunday Service

Jane who is 67 years old is now having to come to terms with empty nest syndrome, after her youngest daughter left the family home 12 months ago. Jane lives in a housing estate in a village located four kilometers from a busy Irish city center. She does not own a car, and given the proximity to the city center, she is able to travel quite easily via public transport. Her activities in the city center are weekly grocery shopping and seeing her grandchildren. Additionally, Jane likes to ride her bike or walk at least twice a week and attends her local place of worship, which she enjoys and has increased her involvement over recent years in because she has the time as well as is growing older. Jane believes it is important to foster and nurture a connection in the community and enjoys a range of activities, including being a member of the local organizing committee to grow organic produce with her friends on their allotments.

Jane admits to not being a regular user of technology; she tends to avoid the use of computers unless it is necessary and is usually assisted by a family member. Jane is a not a smartphone user; therefore, she chooses to stay in contact with friends and family via her home telephone or her basic mobile phone. However, over the last three years, Jane has learnt to send text messages, which has resulted in her growing in confidence. She now finds this engagement as a great way to keep in touch with friends and family. Although technology is a barrier for Jane, but when it comes to providing important communication touchpoints (i.e., contacting family members), she will try to overcome her fears and adapt accordingly. This is true of the recent COVID-19 pandemic situation, whereby Jane and many other citizens, old and young, have experienced limited face-to-face contact with friends, family, and community organizations. Technology has, for Jane, offered an alternative solution to maintaining existing relationships.

**Figure 7:**
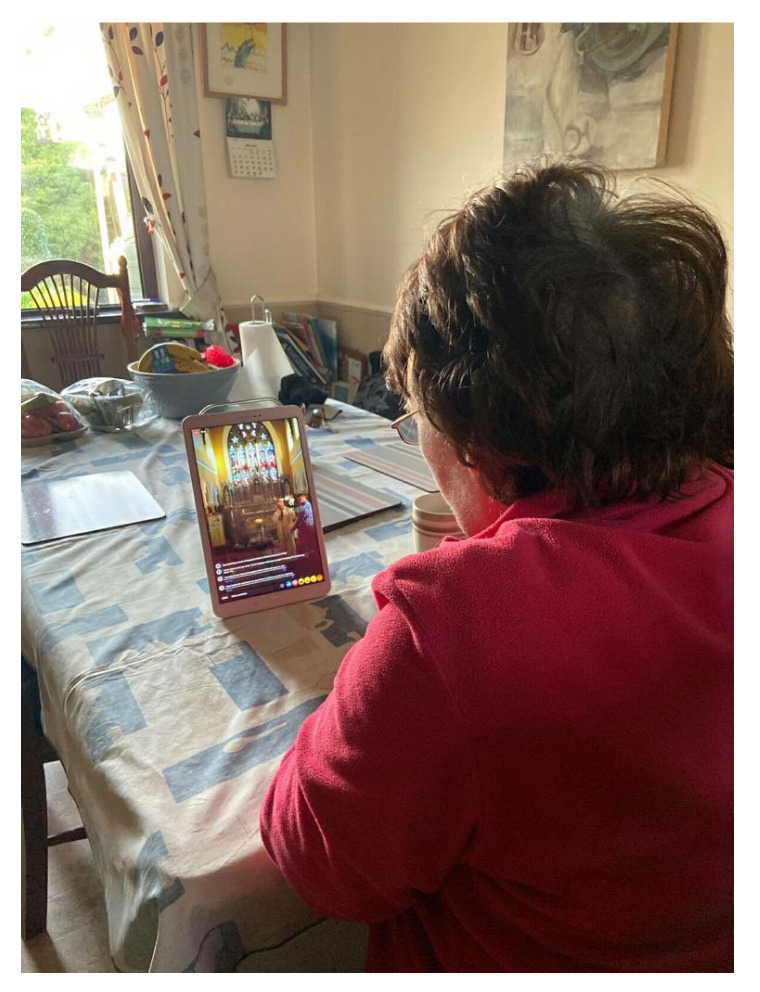
An older lady remotely attending church service in her community.

**Figure 8:**
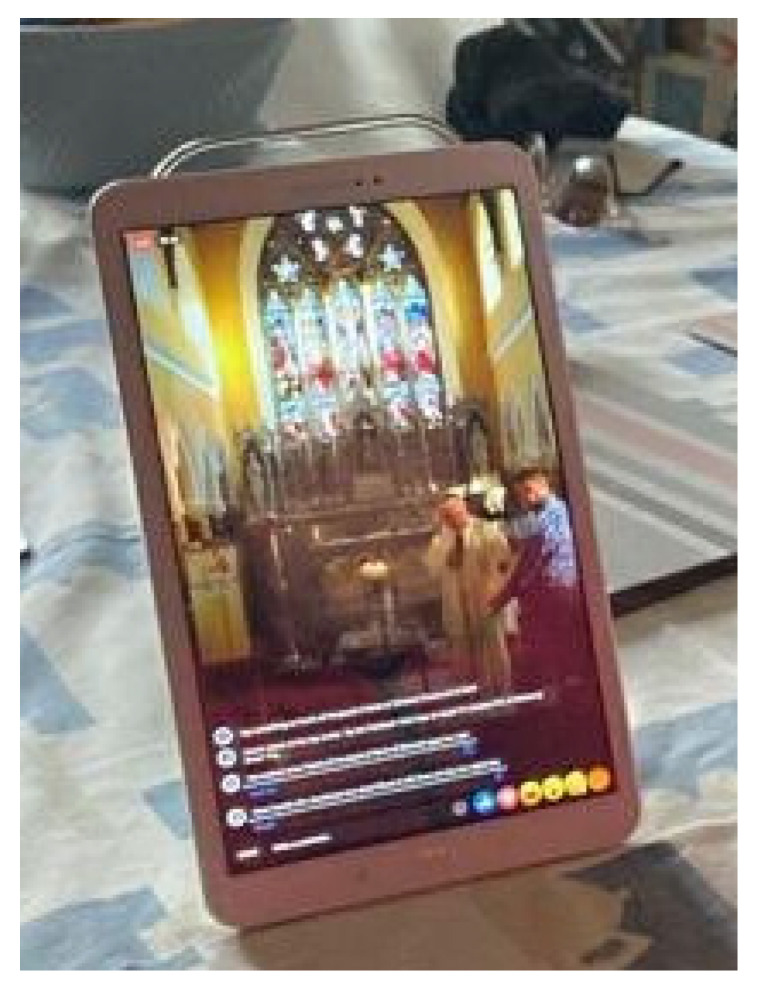
Interface of the church service via Facebook, coupled with comments and emoticons from fellow members of the congregation. Permission granted by Dr P. J. White, taken 2020.

One example of the use of technology as a solution for Jane to maintain her community involvement and friendships is attending the weekly church service Figure 7 and Figure 8). During the first lockdown, there were severe restrictions put in place, which resulted in members of the church not been able to attend their weekly Sunday service. For Jane to attend her weekly service online, her daughter helps set up a tablet device, which in turn presents the church service online church service, while she sits at her kitchen table. Jane accepts that technology in this instance is not a replacement for the church service but admits that it has brought a level of comfort and routine.

There are two important messages, which can be taken and concluded from this adaptation: 1. the perceived barriers of use are overcome with her daughter’s assistance; and 2. the comfort and routine gained from the online church service outweigh her technology fears.

In summary we have described and presented four various case studies that reflect real-world situations, and solutions for older adults who continue to live independently in their own homes. In the next section, we explore several case studies surrounding the COVID-19 pandemic.

## 6. Pandemic Case Studies

From a UK standpoint and context, lockdowns have varied and were introduced into British society on the March 25, 2020 [160]. This approach was taken by the UK Government and is continuing to impact the society from the standpoint of education, economy, mental health, social engagement, and business. Coupled with various societal changes occurring in the 21st century, the UK has been recovering from the 2008 recession, which involved 10 years of austerity, and now with the outbreak of the coronavirus pandemic, which has to date killed 41,988 thousand people in the UK [161], with a further 434,969 cases reported [161].

The week commencing August 10, 2020, the Chancellor of the UK Government (Rishi Sunak) announced [14.08.2020] that the UK was in the biggest recession in 100 years [162], with unemployment reaching approximately 730,000 people [163,164], since the lockdown commenced in March 2020. Furthermore, it is anticipated within a UK context and in conjunction with the furlough scheme ceasing in the autumn of 2020 that the level of unemployment is likely to increase [163,164]. Moreover, citizens and the society does not know what the future holds, as Marston and colleagues note in their blog [29]:

“As the bells struck the stroke of midnight, ringing in 2020, citizens were smiling, pouring another glass of rosé, red wine or supping from their pint of Guinness from the confines of their local pub, house parties, restaurants, or clubs; while singing auld lang syne, shaking each other’s hands, giving a kiss on the cheek to the person next to them or a hearty smooch with a loved one. The biggest challenge of a generation ahead, at this moment in time in the UK was Brexit, little did we know this was about to be surpassed by something even bigger.”

[29]

This is true. At the start of 2020, the Western world could not have imagined a change to their existing ecosystems, daily routines, lives, employment, health, and wellbeing. For many citizens, the use, integration, and acceptance of digital technologies into their individual smart age-friendly ecosystems is the norm, while for many citizens, this is not the case. For many citizens, what was the norm, the regular routines, and expectations of socializing and day-to-day activities, has now been turned upside down.

With regards to these unexpected changes faced and experienced by many citizens, we have provided a variety of scenarios below, building on previous exemplars posited by Marston and van Hoof [30], and in turn represent various ecosystems and sub-groups of populations currently in society.

Scenario #1: Middle-aged family

In contemporary society, it is not uncommon for young and middle-aged adults to have children but also be geographically displaced due to employment commitments which in turn result in having fewer support networks due to their extended families (e.g. grandparents) living elsewhere in the same country or even abroad.

Frederik is a 38-year old man who lives with his partner Zoe, and their two children, Johan (7) and Eva (3). Usually, the children go to school and nursey, respectively, while Zoe and Frederik work. During the week, their home life can be very busy—they have active careers with a limited support network (their families live in the Netherlands). However, their leisure activities are varied, and include playing football for a local team, while Zoe and the children go and watch sometimes. As a family, they like the outdoors—walking, canoeing, and nature. Zoe has recently started exploring the arts and crafts scene, while Johan likes reading, Lego, and watching cartoons with his sister. As a family, they eat freshly cooked meals, and will bake where possible.

The family do not have underlying health conditions, and they usually order their groceries online from a national retailer, while they purchase their fruit and vegetables from the local market stall on a weekly basis.

Since the lockdown, their home environment has changed considerably. They are still expected to work, while the children are not able to attend school/nursery, and Johan’s school is conducting online classes throughout the day. Zoe is wondering how she is going to manage her job, while homeschooling Johan and ensuring Eva is occupied and learning too. Frederik is logging into the computer system so he can continue with his tasks and responsibilities.

Scenario #2: Intergenerational family

In society, there are many families who choose to live together under one roof. Intergenerational living has many benefits, such as sharing household responsibilities, (e.g., caring responsibilities of both young children and older adults), cleaning, cooking, learning, and social interaction. This scenario is rather complex, due to the various generations and situations.

The Smith family currently comprises of three generations living under one roof. They live in the South East of England and due to several changes in circumstances with different members of the family, are now practicing intergenerational living and have been for the last 18 months. The members of the family include Mabel (85 years) and Arthur (90), their daughter Alison (55 ) and son-in-law Stuart (57), who have three children: their daughter Jennifer (28) and her husband Michael (31), their second child Gareth (24), a local entrepreneur and businessman in town, and his girlfriend Sabine (24), who is from Germany, and their youngest daughter Heidi (19), who is supposed to be returning to university in the autumn, in the North of England.

As a family, they enjoy socializing with each other, playing boardgames, BBQing, watching movies, and sport.

Jennifer and Michael are expecting their first child in the summer. Arthur has been diagnosed with dementia and although Mabel is able to care for him, she relies on her family for support. The pandemic has highlighted the vulnerability of both Mabel and Arthur, who are now shielded because of their ages and health conditions, following the guidance and directive by the government.

Both Alison and her husband Stuart are keyworkers, while Stuart is now working shifts.

Gareth and Sabine had been living abroad, and while he had the opportunity to run his multiple businesses from afar, they chose to move back to his parent’s home prior to purchasing their own house. Now that the pandemic has been declared, Sabine assists the family with tasks such as grocery shopping and caring for Mable and Arthur. Gareth has been looking for ways to retain his staff and is considering the furlough scheme.

Heidi is studying at a university in the North of England. She is excited to be returning for her second year of studies but accepts that her second year will be very different to her first. She has been working part-time and will usually transfer her employment to another site when she returns to University. Currently, she is continuing to study her subjects and continue with her assessments; she acknowledges that she is playing a pivotal role in the family ecosystem, alongside Sabine, in caring for her grandparents, and volunteering in the community.

Scenario #3—COVID-19 community support groups

Within weeks of lockdown having been announced in the UK, there were many communities taking an organic approach, by organizing online/social media support groups. Such groups facilitated the community and its residents to assist those most vulnerable or ill with certain requests, such as groceries and collecting prescriptions, among others.

Additionally, specific contacts/residents were also highlighted for streets/areas in that respective community, which in turn would enable a vulnerable resident to directly contact that point of contact and request assistance. This approach was invaluable for those who were shielding/isolating because of illness/health conditions, or who had been diagnosed with the coronavirus.

These support groups facilitated information to be shared relating to opening times, local grocery deliveries (e.g., fruit and vegetables), and regular updates. In some instances, such groups facilitated suggestions to parents who were/are homeschooling children, with the provision of ideas for activities.

Scenario #4—Older adults

Many older adults are continuing to live independently or with their spouses. The notion of death and facing widowhood can be daunting for many older adults, who have spent a significant amount of time together; in some instances, sharing both happy and tough memories and experiences.

The pandemic highlights the vulnerability of older adults in various situations, and this scenario will focus on older adults who are (a) still married, (b) widowed, and (c) live on their own and are aging without children.

Older adults—still married: Margaret (67 years) and Stan (70) have been married for 50 years; they have two children, six grandchildren, and 1 great-grandchild. They survive on their state pensions, and Stan has a small private pension. Their children (Kathryn—49 years, and Martin 47 years visit regularly throughout the week, popping in for a cup of tea and a catchup. The grandchildren range between 17 and 25 years; their great-grandchild is one year old.

Older adults—widowed: Derrick is 65 years old and recently widowed. His wife Stella died 8 months ago from a neurological condition at the age of 62 years. Derrick is adjusting to life on his own; he has a daughter who lives approximately 2 hours away, and who tries to visit her father on the weekend. Derrick still has friends and tried to maintain the odd social event at his local club, where he could still interact.

Older adults—live on their own: Suzanne is a 57-year old woman who recently took a severance package from her employer. She has never married, is aging without children, and her extended family are scattered across the UK and abroad. She enjoys cooking, growing vegetables, volunteering at the local charity shop and church, and arts and crafts.

Scenario #5—Resident/assisted living/care home(s)

The pandemic has cruelly highlighted the vulnerability of citizens residing in environments such as residential, assisted living, and/or nursing/care homes [165]. Early in the pandemic across the UK, it was reported that many staff (e.g., carers, chefs, management) chose to live in, in the respective home to 1. shield the residents, 2. reduce the risk of catching COVID-19 and passing it on to colleagues and residents, 3. similar to point two, but passing the coronavirus on to their families [166]. How will such environments operate in a post-pandemic society from the standpoint of carers/support staff, residents, and family members?

Scenario #6—Young person living on their own

There are many young people who move away from their friends and family for employment and/or studies. The pandemic is highlighting social isolation and loneliness, and mental health issues are impacting not only older adults, but young people as well.

Carl is a thirty-something professional who lives in a different County, approximately 6 hours from where he grew up. He has been living alone, renting an apartment in this area for some time but has found it difficult to form a solid friendship/social network. One of the reasons for this is because Carl has been on fixed-term contracts with his employer, which has hindered his ability to make friendships in the town. Carl’s personality is outgoing—he enjoys watching sports, enjoys his job, and runs regularly with a running club. However, he has tried to form a social network through the running club, work colleagues, and his neighbors, but to no avail. Carl usually spends his down time reading, watching Netflix, and cooking.

Scenario #7—Family who has a member with serious health condition(s)

For many citizens who themselves suffer from or have family members with serious health conditions—cancer or a life-limiting or life-threatening health condition—the pandemic has added additional pressures to their home environment.

Darren (35) and his wife Roberta (33) have two children, James (7) and Amelie (11). Darren has been in the military for 15 years, while Roberta usually works part-time in the community. They live off base, which affords the family a greater level of freedom and gives them the opportunity to socialize with friends and colleagues on the base, enjoy BBQs, annual parties, and other events. Amelie has just finished junior school and is starting high school in September; she is looking forward to this transition and enjoys learning and making new friends.

James has a life-limiting health condition, which was diagnosed when he was toddler. Due to his health condition, Roberta attends various meetings with health and social care practitioners to ensure all his needs are being met, and his progress is recorded. Darren attends when he can; due to his work responsibilities, he finds it difficult to attend all the appointments and this at times means Roberta is caring for their children as a single parent. Although she has a social network in the community and amongst friends on the military base, their familial support networks are geographically displaced because her and Darren’s parents live several hours away.

Since the pandemic and during lockdown, Darren has been away on exercise, which has led Roberta to be on her own, homeschooling the children, attending to the physio exercises for James, and furloughed from her job in the community. Her social interaction has thus been reduced considerably, and due to James’s health condition, she and Amelie have had to shield themselves. Their friends from the military base have come by, dropping groceries off, and chatting through the window. Usually, Roberta is not troubled by the absence of Darren, because they have been together for over 10 years, and when they married, she knew this would be part of their life. Roberta communicates with hers and Darren’s parents, but she is experiencing the additional stresses of homeschooling, social isolation, and loneliness while Darren is away, which is exacerbated by James’ health condition and the pandemic. Appointments that had been scheduled for several months/weeks are not taking place or have been reduced.

These scenarios aim to illustrate the different circumstances of citizens across various age cohorts and home environments, in an attempt to reflect the everyday life for many citizens. Playing out such scenarios offers greater opportunities in identifying what type of technology, IoTs, support, and research can be roleplayed.

Taking into account the scenarios presented above, we propose and discuss the ‘Concept of Age-friendly Smart Ecologies (CASE)’ framework in the following section. The CASE framework is built on the WHO [36] framework and the extended framework by Marston and van Hoof [30].

## 7. Proposal of New Post-Pandemic Age-Friendly Ecosystems

**Figure 9:**
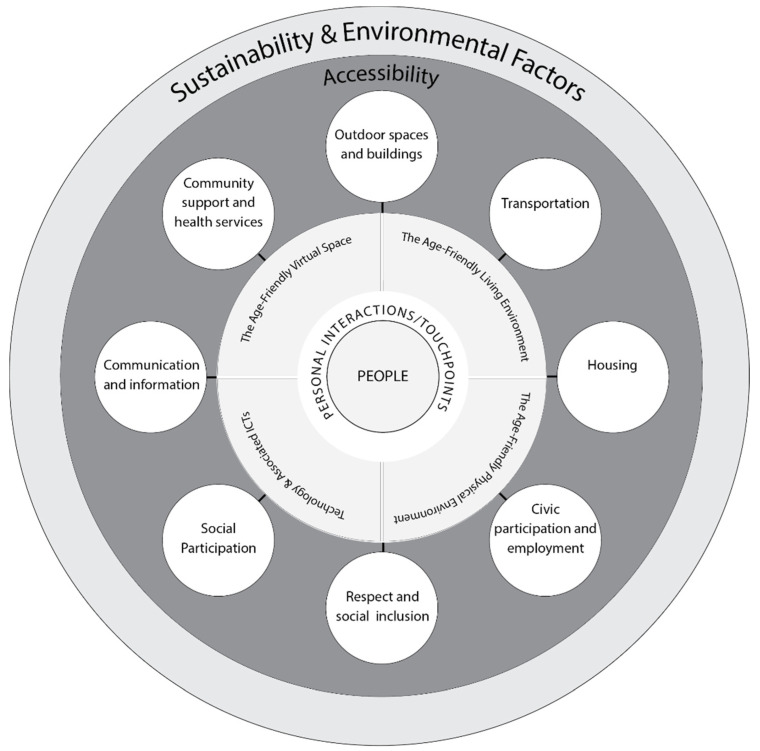
The newly proposed ‘Concept of Age-friendly Smart Ecologies (CASE)’ framework.

Based on contemporary literature surrounding existing age-friendly frameworks [30,36], in conjunction with earlier discussions posited in this paper (i.e., design hacks, and the various case studies and scenarios), we believe a new ‘Concept of Age-friendly Smart Ecologies (CASE)’ framework (Figure 9) can be proposed to offer a myriad of actors the opportunity to adapt the ‘Concept of Age-friendly Smart Ecologies (CASE)’ framework to suit their needs and requirements, or to various situations.

Furthermore, taking into consideration the rapid pace at which technology develops while ensuring all citizens in society are represented, the coining and positing of this term ‘Concept of Age-friendly Smart Ecologies (CASE)’ offers a myriad of actors the flexibility to adapt and future proof respective environments, where necessary.

The ‘Concept of Age-friendly Smart Ecologies (CASE)’ framework is an extension of the Marston and van Hoof [30] and WHO [36] frameworks. 

In the CASE framework, we added several new sections to reflect on and consider existing as well as new areas of interest. The CASE framework is as follows: 1. The outer sphere is ‘*Sustainability, and Environmental Factors*’ which relates to ensuring that all citizens, companies, organizations, educational institutions, etc. can and are contributing to a greener, more efficient, and sustainable environment. This could relate to local, regional, and national environments. This sphere can aid policy makers in assisting with this transition by including not only environmental benefits to adoption of sustainable approaches, but also economic advantage [166], which can initially appear high, but over time reduces. This, in turn, can benefit economies and budgets. Furthermore, by taking a co-creation approach in conjunction with a universal design approach, this outer sphere facilitates a wide variety of opportunities for many actors to co-design, co-create, and co-produce existing physical environments.

2. *Accessibility* is reflected by the inner sphere and relates to the accessibility of the intersection occurring between the relationships of the physical and digital ecosystems conducted and experienced by citizens, business, educational institutes, community hubs, etc. Because accessibility relates to and is interwoven through differing features of the infrastructure within our cities or towns, it is important that there is a strong relationship and understanding of how accessibility intersects our daily lives. Accessibility is incorporated through (a) buildings and transport, (b) business, educational institutes and community hubs and services, (c) packaging (including pills/medications), and (d) technology. This in turn is a necessary consideration to facilitate and adapt to the age-friendly concept for all citizens. It is apparent in the previous age-friendly framework [30] that accessibility is an important factor for all eight hubs, highlighted in case studies discussed in this article and illustrating how citizens can be hurt by poor accessibility design considerations. 

3. The eight hubs represent various aspects in society and are taken from the 2007 WHO age-friendly framework [36]. 

4. As we become more technologically immersed in the current and future experiences of our respective ecosystems, both in contemporary and future societies, technology will continue to play a key role. The CASE framework ensures a notion of future-proofing the approach to design considerations of smart age-friendly cities by introducing four separate sections in the inner sphere, which are explained in Section 7.1 and highlight the beneficial outputs and scenarios to: (a) The *Age-Friendly Virtual Space*, (b) The *Age-Friendly Living Environment*, (c) The *Age-Friendly Physical Environment*, and (d) *Technology and Associated ICTs*.

5. The inner hub—*Personal Interactions/Touchpoints*—represents interactions and experiences that can be personal or shared with an individual. In contemporary society both pre-and-during COVID-19, many citizens are experiencing loneliness and social isolation because of both geographically displaced families as well as governmental lockdowns or cocooning [62]. The purpose of this inner hub aims is to afford citizens the emotional and social needs that are central to positive health, wellbeing, and age-in-place.

6. The central hub represents the citizens in both our current and future societies. Acknowledging the needs of citizens needs to remain central in ensuring that all that is offered, through various interactions within respective environments and operating systems, and with the use of products, has the potential to offer positive experiences for all—both young and old citizens. Furthermore, it is intended within the inner hub that there will be support afforded via human interventions and supportive networks and systems to those in need.

Indeed, while we are discussing the CASE framework from the standpoint of contemporary society, we should also take into consideration the future and what or how society will look in a post-pandemic society. While the *CASE* framework can reflect existing societal mechanisms, would differences occur for several actors relating to interaction and engagement at various micro and macro levels in respective ecosystems, which in turn may afford greater agility, adaptability, and scalability [70,124]?

For example, in a city, this diagram represents a district or a suburb depending on the size of each of the eight hubs: 1. Transport, 2. Housing, 3. Civic participation and employment, 4. Respect and social inclusion, 5. Social Participation, 6. Communication and information, 7. Community support and health services, and 8. Outdoor spaces and buildings.

The size of each of these hubs or domains may vary based on the positive and negative impacts indicative within each district or suburb. Similarly, this notion could be adapted and scaled up to reflect municipalities or provincial regions within countries. Therefore, the greater is the hub associated to one of these eight domains, the greater is the positivity or service(s) offered (e.g., public transport, ICT infrastructure, health service provision etc.). Alternatively, the smaller the hub, the lesser or negativity of services are afforded.

In summary, the *CASE* framework displays an innovative approach to amalgamating both the WHO [36] and SAfE [30] frameworks, while also recognizing that there are additional factors at play within the ecosystem that were not implemented or acknowledged in previous iterations. Furthermore, the *CASE* framework affords various actors the opportunity to evaluate their respective ecosystems given the increase in technologies and the potential changes and behaviors in the future, in a post-pandemic society.

In the following section, we describe a series of scenarios and provide solutions based on point 4 of the *CASE* framework.

### 7.1. Design Hacks, Technology, and IoT Solutions

In this section, we will revisit the different scenarios described in the previous section and suggest suitable solutions.

Scenario #1—Middle-aged family

Regarding scenario 1, the *CASE* framework accommodates the personal interactions that everyone has, in this instance, in conjunction with other family members (e.g., partner to partner, parent to children and them to their parents). In both a pre-and-post-pandemic society, the relationship between the user, technology, and the environments involves lived experiences within the eight domains. However, the new *CASE* framework central quadrants provide an insight into potential and specific interactions and touchpoints to these domains.

The Age-friendly Living Environment: As this family has not experienced aging, it may seem strange to explore age-friendly environments. However, the living spaces we reside in, both currently and in the future, may be modular or adaptable spaces that can consider areas for collective calm—they include features based on soundproofing and adequate sensory ambience, which in turn can encourage relaxation and to recharge oneself while facing the challenges of living through both a contemporary and post-pandemic society. Additionally, all family members can benefit from these spaces and there can be a shared space too, in addition to family members retreating to their bedrooms. Growing up in this type of space, children may have the opportunity to perceive this space as a form of relaxation as a part of a daily ritual, while learning valuable means to relax in stressful or worrying times.

The Age-friendly Physical Space offers this family the option of outdoor sharing or single activities, such as walking, canoeing, and enjoying the experience of time surrounded by nature. However, beyond these experiences, life in both a contemporary and post-pandemic society continues to have the additional requirements, such as shopping, waste, and health management.

This brings to the fore the possibility of sustainability and environmental features. It is apparent that people’s view on fast fashion has changed to exploring ‘slow fashion’ or repurposing clothing, furniture, and sharing economies [167,168]. Zoe’s interest in the arts and crafts scene endorses this and creates an opportunity to create items for the home, as gifts, or garments to wear. ‘Crafting’ is a hobby that is shared and interweaves generations, and an age-friendly community network of crafters was visited pre-pandemic [158]. Networks such as this can reopen in a post-pandemic society. However, visitors will be presented with hygiene and virus management challenges, ensuring each member is protected. To overcome this, there is the possibility of ensuring that materials are maintained and not shared, unless appropriately sanitized. These community craft times could perhaps provide greater comfort and opportunity for parents such as Zoe to attend alone or encourage a smaller group of children to explore various art-and-craft activities. There is also the possibility of intergenerational activities, whereby similar to the old ‘punch and Judy’ model, the players are behind the scenes and the audience can safely socially distance in new open spaces in towns, cities, or parks.

The Age-friendly Virtual Space is an exciting space to speculate a post-pandemic society. The possible solutions both encourage and include interactions with people and families with mobility or physical limitation. Relating to scenario one, the solution could offer interactive augmented reality spaces for education and play time for Johan and Eva. These spaces could be pods similar to how children played in a pre-pandemic society, whereby locations such as ball pit, play zones or trampolines and playgrounds facilitated free play.

Technology and Associated ICTs: this scenario considers the competencies of both Frederick and Zoe, who have busy lives, careers, and personal ambitions. In this case, it is imperative that various service systems, for example, health, energy, and home/banking management, can operate in a usable, friendly efficient way. Service providers have adapted greatly by increasing offerings, such as online shopping for groceries or financial transactions becoming increasingly contactless payment. Considering the post-pandemic world, some of these new behaviors will be maintained (e.g., contactless payment, increased from £30 to £45 in the UK). However, new forms of behavior, such as hybrid working—encompassing greater affordances and approaches to work from home—will rely on existing and innovative technologies to be accessible, and not being restricted due to network or server issues and difficulties.

Scenario #2—Intergenerational family

The Age-friendly Living Environment: The intergenerational family outlined in this paper has multiple needs for the current living environment they share. In a post-pandemic society, it is apparent that there is going to more changes and it is likely that Gareth and Sabine will seek to move home, while Heidi is anticipating her return to college in the autumn or experience a mix of online and face-to-face teaching.

The future experiences for Gareth and Sabine are normal events for most families as they grow. In a post-pandemic society, should a request for ‘shielding’ or ‘cocooning’ be advised, they may also include home adaptability or receive new responsibilities of care and support. Perhaps, not so much that Arthur and Mabel isolate, but for keyworkers such as Alison and Stuart to be reassured that they are able to continue their work, yet taking into account that they will not present any greater risk to the members of their own home environment.

It is suggested that perhaps an entry point is agreed in the home with a specific space for sanitizing, undressing, and showering to reduce the risk of spreading the virus to vulnerable members of their own household. Sabine appears to be at home to provide care and support, but this may change as the baby is born and how this will affect the family dynamic and use of comfort and spaces is unknown.

As per scenario 1, one solution could be to live in a modular housing development/unit, which in turn could support this family to allow for temporary move-ins (Gareth and Sabine) or connected living spaces that present a gathering opportunity or shielding for Arthur and Mabel.

The Age-friendly Physical Space: Accessibility has been noted as a feature within the domains of the age-friendly network (outdoor spaces and buildings). Health conditions and lifespan events (e.g., giving birth) can impact how we move from one location to another.

Currently, and during the lockdown in the UK and Ireland, public transport was restricted because of limitations on the numbers of passengers or services operating. As the months continued, users of public transport had to comply with governmental legislation relating to facemasks. Should a person wish to use public transport, they are required to wear a facemask. In a post-pandemic society, envisioning how society will behave could afford municipalities, governments, academics the opportunity to purvey alternative forms of transport/hubs. For example, the use of autonomous vehicles, bicycles programs (as found in Copenhagen) or shared transport networks could prove to be beneficial, because this would facilitate health and wellbeing, physical activity, and reduce the risk of any further contagion. 

The Age-friendly Virtual Space can have a positive impact on connected health developments, such as interactive spaces between healthcare professionals or care workers and patients such as Mabel and Arthur. Likewise, additional connectedness could be experienced from within the home ecosystem by differing technologies, such as virtual assistants, alert systems, or apps accessible and even controlled remotely via a smartphone. This, in turn, would offer reassurance to family members if a vulnerable family member is alone, or should assistance be required, they can respond quickly.

Technology and Associated ICTs can be associated and connected to both healthcare and transport hubs and links, which, in turn, could provide supportive networks that facilitate and enable the autonomy and responsibility of lifestyle and independence. The monitoring of symptoms and testing facilitated by track-and-trace capabilities during the pandemic can be potentially done in a post-pandemic society. Furthermore, the integration of Blockchain and AI capabilities has the potential to afford healthcare providers, municipalities, business, and citizens the opportunity to engage both directly and indirectly within respective ecosystems. Additionally, promotions or campaigns across various modes of digital devices, social media platforms, and physical spaces (e.g., advertisement boards) can assist in reminding citizens to maintain regular hygiene practices, which, in turn, may reduce the opportunity of further susceptibility in a post-pandemic society. The recent progress made in Africa whereby the elimination of polio has progressed [169] is a good example of this.

Scenario #3—COVID-19 community support groups

Scenario #3 explores the activism conducted by people within communities to be supportive and helpful to those with needs such as those ‘shielding’ or in fact frontline workers who may have very little personal time to refresh.

The Age-friendly Living Environment: For an individual living alone or deemed vulnerable, they may still have requirements and needs that must be supported by neighbors or people within the community. Apps or simply creating a WhatsApp/phone group that supports images, videos/video conferencing, and voice share could continue to assist vulnerable people in a post-pandemic society. Likewise, the mobilization of smaller factories responded rapidly to the call for clothing to be worn by frontline workers should be maintained as a network that could be mobilized, should there be another outbreak of COVID-19 or another coronavirus/ emergency.

The Age-friendly Physical Space: Community ‘hubs’ could be encouraged and implemented by either utilizing existing structures such as community halls or creating new purpose built hubs, which, in turn, could provide leisure activities and work spaces, but also double up as an emergency area, should similar pandemics occur in the future.

The Age-friendly Virtual Space: Virtual and interactive opportunities could be made possible by applying AR in conjunction with social media and communication platforms, to enable and ensure group activities, as well as the delivery of education, health, and business meetings, are continued. This would be fruitful because if all members of the respective outlets are not available to attend in person, they can still attend virtually.

Technology and Associated ICTs can be implemented in community and connected activities to enable reliance on existing hardware and devices such as mobile phones and computer applications, which, in turn, will support contacts and updates to mobilize or stand down, should there be emergencies in the future. One prospective solution is to create a volunteer registry, which would facilitate and reassure each user/member of the security of their information and personal details; one that is transparent but yet, easily accessible via instant messaging, to ensure accessibility for all.

Scenario #4—Older adults living independently

The Age-friendly Living Environment relates to age-in-place [170,171] and is perceived as a beneficial approach to aging in later life, with a view to building on and sharing positive aging experiences [159].

Living independently highlights the opportunity to explore the home environment with a view on accessibility, which, in turn, could be easily adaptable, if necessary. Furthermore, new homes could be built with the view for positive and successful age-in-place, whereby doorways are wheelchair-friendly, light switches are placed at an accessible height, rather than at a height for a person standing up. Staircases and landings on the first floor are to be of suitable width, which enables wheelchair access and/or mechanical stairlifts to successfully transfer an individual from the ground floor without ruining the decoration. These approaches could be considered by William and his wife – as discussed in Case Study C. Luckily, there are building recommendations that support accessible a universal design in new builds, and retro fitting grants to adapt homes, typically after a need (e.g., home access ramps) is identified, can be made available. Additionally, we would suggest further features that support autonomy and security in the home. Whether from the standpoint of the pandemic or a post-pandemic society, the consideration of built environments to support a form of socializing but still maintain shielding could be explored in future developments [149]. Finally, what has been highlighted here is the essence of greater opportunities for the construction industry, developers, planners, architects, and academics in the fields of gerontology, gerontechnology, social sciences, and HCI. This could take a co-creation, co-design, and universal design approach to understanding the needs, challenges, issues, experiences, as well as positives of this type of living and development [62].

The Age-friendly Physical Space: Maintaining social connections appears to be a significant factor for older adults living independently and who are ageing without children (AWOC) [29]. This can be emphasized in a post-pandemic society by more activities that can be conducted in public spaces, such as green spaces, streets, community hubs, or gyms. Additionally, there could be the option for intergenerational spaces, which, in turn, could cater to all ages; they could have giant chess, boule, and table tennis tables. Implementing sensory spaces could afford residents and citizens the opportunity to relax outdoors, whereby seating is surrounded by different sounds, images, and touch and scent of the flora and fauna within the space. These spaces would include energizing areas that capture sunshine and places that are more serene and shaded.

The Age-friendly Virtual Space relates to various activities and energy we have, and which can change during the aging process. We may develop new health conditions (e.g., arthritis) or experience more severe and impactful diseases, such as a stroke or a heart problem. Recuperation and recovery programs in a post-pandemic society may be a feature of a new connected health service, and interactive screenings and appointments could be considered by municipalities or local health care providers.

Technology and Associated ICTs relates to maintaining one’s independence as we age and move forward in a post-pandemic society, while creating new opportunities to explore robotic assistive devices that can enhance the independence and autonomy of an individual. IoTs and wearable technologies can provide reassurance (e.g., monitoring or alerts to falls, or sudden increase in body temperature) to neighbors, family members, and friends of the individual. These types of sensors and devices can also relate to alternative, new connected health programs that includes relevant professional and trusted members of an older adults’ network (e.g., support network, health professionals, family, friends etc.). Blockchain and AI technologies have the potential to offer this type of service delivery, with focus on data privacy and security.

Scenario #5—Resident/Assisted living/Care Home(s)

The Age-friendly Living Environment: This scenario is similar to intergenerational living [62] and their respective needs. However, unlike an intergenerational family, bonds may not be emotionally strong. An example of this could relate to the care assistant or nurse, who choose to be a live-in, and who may also still be juggling her own family responsibilities, albeit remotely. Therefore, the resident/care home may require the assurance of staff calm spaces, where they can adequately support social interactions with other staff members or likewise have zones of relaxation and calm, where energies can be renewed.

The Age-friendly Physical Space, from a post-pandemic standpoint associated to residents of care homes, may renew shopping trips or outings that involve groups or sometimes outings with family that were enjoyed in a pre-pandemic society.

It is apparent that while there is no vaccine for COVID-19, it is not yet stated how long society will be continuing with differing variations of lockdowns and legislation. Therefore, wearing facemasks and using hand sanitization will become integral in day-to-day rituals (e.g., going into a grocery store, touching public artifacts, etc.).When we consider some aging factors, such as reduced hearing, vision, and ambulation, facemasks may present a challenge, not just in how they are worn, but also potentially interfering with hearing aids and/or glasses. Additionally, a facemask may also have an impact on the proprioception and/or spatial awareness of a person within the physical space, and this in turn may lead to them losing their balance and falling/tripping over. In turn, this requires assurances that features such as lighting or access is optimized to cater for all abilities and citizens.

The Age-friendly Virtual Space is important for the residents and staff of residential facilities and care homes. Technology for some of these facilities may be limited, coupled with the digital skills/literacy of the staff. However, technology and appropriate broadband networks are needed to ensure that features such as video calls or classes can still be conducted and experienced via communication platforms, which also facilitate a virtual space to socialize and engage with community activities, such as attending church services, as demonstrated in Case Study D, Section 5.1.4.

Technology and Associated ICTs can assist residents and staff in care homes with maintaining social and familial connections by taking a deep dive into technology and using the various features accessible in different social media and communication platforms, such as video calling, looking at photographs, listening and watching music and television programs, as well as communicating via email. Voice assistants may be helpful to provide social engagements for residents who are alone and may need to alert a member of staff for assistance. Additionally, with virtual assistants, there is the potential to be connected to wearable devices, which may also offer a feeling of safety to the resident and their family members.

Scenario #6—Young person living on their own

The Age-friendly Living Environment: For Carl, who is a healthy and fit person, this environment in both a contemporary and post-pandemic society may afford greater opportunities to enhance interactions that have been ‘held back’ due to geographic distances, detached meetings, and social outings. 

The living environment has at times offered little comfort to people like Carl, who experience loneliness and social isolation. A suggested enhancement to the living environment would be greater accessibility on a long-term lease, which, in turn, would facilitate someone like Carl to feel ‘at home’ or ‘in place’. The possibility to ‘embed’ or feel at home could build and enhance confidence to pursue more robust friendships or social networking opportunities through the living and/or communal spaces within these new environments. There might be a choice to have a ‘pet’ that does not require full responsibility of one person but offering a ‘pet share’ plan could enhance further social interactions with the partners of the pet.

The Age-friendly Physical Space lends itself to home working pods that are not coffee houses or linked with commercial brokers, but instead could be developed in a way that could retrofit unused or redundant spaces in localities. This type of example could work for neighbors who may be working from home, but could then congregate in a mimicked work environment, a ‘Work-Gym’. Furthermore, this concept may also encourage new friendships, relationships, and broaden social networks.

The Age-friendly Virtual Space could support working from any geographical location; for Carl, this could mean he remains living in his hometown, surrounded by all things familiar, whilst working remotely, connecting through digital applications to engage in meetings or updates regarding projects or team collaborations. Alternatively, should Carl choose to work or relocate to a new geographical location, he could mirror certain behaviors, experiences, and views from home through ambient and responsive AR scenarios.

Technology and Associated ICTs can include virtual assistants and social robotic pets to enhance one’s quality of living experience although it would not be a replacement for face-to-face contact. This proposal encourages a ‘kit’ whereby you build and include your preferences to personalize the type of home you wish, and the robotic pet could, for example, be a replica of a childhood pet. The benefits to people like Carl are the freedom to still take trips or visits to his hometown without planning or having the responsibility to find a suitable pet sitter or the cost of kennels.

Scenario #7—Family who has a member with serious health condition(s)

The Age-friendly Living Environment: More so than ever, people have experienced various impacts and difficulties during COVID-19. Family life can be challenging during normal times and it is apparent how Darren and Roberta rely on social scaffolding to enable a positive quality of life, socializing and interacting with friends and members of the community.

The work responsibilities Darren has impacts on Roberta’s ability to make sense of day-to-day family living and time management. Additional support to the family could be further respite care for James, which allows for moments of refresh for Roberta, particularly when Darren is away. However, during the pandemic, this opportunity may not be possible. However, as James grows up and his needs become more complex, a respite facility may afford all family members the opportunity to relax. Furthermore, identifying appropriate networks within their existing social and familial networks and organizations could afford Roberta the opportunity to take time out for a walk in the green space close to their home and enjoy some time with their daughter Amalie before she starts high school. One of the support networks could assist by taking over homeschooling duty with James. This system could perhaps be encouraged and reversed during term time (e.g., one-to-one time with James, while a member of the support network homeschools Amalie).

The Age-friendly Physical Space: During the initial lockdown in the UK, there was a regular/weekly clap for frontline workers every Thursday evening at 7 pm. This was an important action to acknowledge all those citizens who were and continue to work on the frontline (e.g., medical professionals, health and social care providers, etc.) who are compromising their own health, their time with family, and personal lives to support those who fell ill.

Perhaps in a post-pandemic society, there could a way we should look at ways of remembering to take time to value those close to us and state it in a subtle way. Instead of purchasing items of ‘stuff’, it could simply be a gesture that is on a physical living space in a neighborhood. A thank you wall/park/space might work, whereby an assigned space affords the provision to allow for blocks or ornaments to be attached to this space to be purchased on behalf of someone by another person. This space would be accessible to all and could be shared and offer a space for reflection, while focusing on the gratitude for those individuals and keyworkers who served their communities during this time. This essentially could be a funded arts project that could be updated over time but is always changing and reflective of where life might be at that given time.

The Age-friendly Virtual Space could offer families and couples such as Darren and Roberta a specific space to spend some quality time together, while also being connected to specific health technologies. This, in turn, would allow healthcare professionals, patients, and family members to interact and share progress or prognosis updates securely, while not necessarily being together in any specific location. Sharing of information would be facilitated via the implementation of Blockchain and AI solutions, and securely accessed via an App and/or via a communications platform, or virtual assistant. Additionally, wearable devices and analytics could offer efficient updates and insights in real-time for James’ parents, and his healthcare team. The design outcomes that are desirable in this prospective solution relate to data security, comfort, and ease of use for all key people involved in the health, wellbeing, and service delivery for James.

Technology and Associated ICTs: Accessibility and ease of use of service systems are necessary aspects to gain fully functioning optimization. Technology requirements would need to be supported on secure networks (e.g., via Blockchain) and future-proofed to ensure that the lifespan of the design for patients such as James is maintained and updated, where necessary. Additionally, the family could engage and manually update the system with personal additions/observations to the health system ‘App’, thereby allowing for random or surprise changes to James’ prognosis to be captured. If Darren is away with work, he can still access, view, or add thoughts relating to the information presented on the App.

In summary, this section has provided possible solutions to the differing scenarios presented in Section 6 and relates to the four quadrants of the *CASE* framework. These theoretical solutions afford readers the opportunity envisage how different technological solutions could be implemented into different ecosystems.

In the following section, we discuss the work presented in this paper and provide our recommendations, strengths, and limitations.

## 8. Recommendations and Conclusions

This paper proposes an innovative smart age-friendly framework for existing sites that house previously purpose-built buildings and institutions, which, historically as well as currently, serve citizens from across the region and further afield.

Building on an existing model by the WHO [36], and the extended model proposed by Marston and van Hoof [30], we proposed an alternative framework called the ‘Concept of Age-friendly Smart Ecologies (CASE)’, which considers an age-friendly society, taking both a contemporary and post-pandemic approach for citizens in the Western world. The various scenarios and case studies illustrate real scenarios that citizens are currently experiencing. Marston and van Hoof [30] stated in their extended ‘Smart Age-friendly Ecosystem’ (SAfE):

“Within and across society, and the lives of citizens, the relationships and engagement between the central, inner, and outer hubs/spheres will vary, based on users’ needs, expectations, access to services, facilities and amenities. Sharing information via a closed, select group of friends/acquaintances is not unfamiliar and offers members of that group the opportunity to share information in real time and very quickly.”

(p.26)

With this in mind, the ‘Concept of Age-friendly Smart Ecologies (CASE)’ greatly expands this notion, whereby we have integrated and added additional spheres, hubs, and segments to represent engagement within various ecosystems by citizens and different actors at various intervals within the ecosystem, depending upon the activity and rationale.

Limitations of this proposed work and framework is the lack of qualitative and quantitative data to support the ‘Concept of Age-friendly Smart Ecologies (CASE)’ framework across society and its lifespan. The proposed *CASE* framework is theoretical and aims to reach out to multiple actors who are interested in transforming existing towns and cities into Smart Age-friendly Ecosystems (SafE) [30]. Evaluation of the *CASE* framework is needed, and this could be conducted through a living lab approach, as described by Shin [70], Shin and Park [124], while implementing universal design principles to evaluate the framework. These principles would facilitate the evaluation of future case-studies with a defined metric. The newly published ‘Design for All’ standard I.S. EN 17161:2019 [172] has appropriate measures to help commence this research.

The strengths of the ‘Concept of Age-friendly Smart Ecologies (CASE)’ framework includes the opportunity for towns, regions/counties, provinces, and municipalities to take an agile approach and work together in a locality approach to adopt and implement improvements aimed at making their existing environments into a SAfE, which, in turn, could afford residents and citizens greater benefits across the various hubs and infrastructures, by employing innovative technologies, such as Blockchain, AI, EVs, and digital portals and Apps.

Furthermore, these changes could be in the form of parish councils, regional councils, or from the perspective of the UK, regional mayors such as the position in Manchester—where at present, the Mayor of Manchester is a former member of parliament (MP)—Andy Burnham [173]. Additionally, we believe that this proposed ‘Concept of Age-friendly Smart Ecologies (CASE)’ framework is timely, given the global pandemic and how citizens in society, businesses, educational institutes, and health services are currently having to adapt and work in a more agile approach. At present, we do not know how long this pandemic will continue, and with this in mind, we have structured solutions in Section 7.1 from both the perspectives of contemporary society as well as looking into the future—in a post-pandemic society.

This position paper opened up with the narrative of the last epidemic and pandemics experienced in the 20th century, and it is possible that this pandemic of the 21st century will be around for some time, maybe forever, or until a vaccine is found. Either way, citizens globally are now having to adapt to the day-to-day changes and expectations set out by respective governments, based on the science of respective government advisors such as the SAGE (Scientific evidence supporting the government response to coronavirus (COVID-19)) committee for the UK/England [26].

At the time of writing this paper, the UK has various directives set out by respective devolved governments (Wales, Scotland, Northern Ireland, and England). As of 17–18 September 2020, the region of the North East (i.e., Northumberland, North and South Tyneside, Newcastle-upon-Tyne, Gateshead, Sunderland and County Durham) [174,175,176] and the North West (i.e., Lancashire, Merseyside, and Warrington) [177] of England have been informed of greater sanctions, which include a curfew between 10 p.m. and 5 a.m.—pubs are to be closed by this time and households are not allowed to mix and socialize outside of their social bubble/household [176,177], because of the rise of recorded COVID-19 cases.

Furthermore, from Tuesday 22 September 2020, additional regional lockdowns have been imposed across the Midlands, West Yorkshire, and further areas of the North West [177]. In Wales, the areas of the Rhondda Cynon Taf (RCT) and Caerphilly counties have had additional restrictions imposed on the citizens. Residents in the RCT area not permitted to enter or leave the area without a valid reason, facemasks must be worn indoors in public spaces, households are not allowed to continue socializing in their extended bubbles, and meet-ups must be outside and all pubs should be closed by 11 p.m. (1 h difference for England) [178].

For residents who have family members living in care homes, visits are now suspended in these respective areas, acting as a precautionary measure to keep all care home residents safe [179]. Yet, from the standpoint of Northern Ireland, the areas of Ballymeana, postcode areas of Belfast (BT43, BT28 and BT29—Glenavy, Lisburn and Crumlin) face restrictions on visiting friends and family indoors, and no more than six people from two different households can meet in a private garden. While essential visits to care homes and hospitals are permitted, there are restrictions on the number of visitors, and it is likely these tighter restrictions will be in place for two weeks [180].

Additionally, another area of Northern Ireland—BT60—which covers areas of the County of Armagh will have sanctions imposed from 5 p.m. on Friday 18 September 2020 [181]. Northern Ireland will commence ‘drink-only’ pubs from 23 September and this includes no dancing, table service only, only six people are allowed to sit at the same table as long as they are from the same household, track and trace (all customers to provide contact details), and face masks should be worn on entering/leaving the premises [181]. There is no restriction to closing time, unlike England (10 p.m.) and Wales (11 p.m.). From the standpoint of Scotland [182], indoor spaces that facilitate soft play, theatres, live music, and contact sports for young people aged 12+ years are prohibited from reopening until 5 October.

Residents in Scotland can meet both indoors and outdoors to a maximum of six people from two different households, while there are exceptions for specific events (e.g., funerals, weddings, organized sports, and civil partnerships), and physical spaces such as places of worship, which include a maximum of 20 people, been allowed to attend receptions and wakes at venues (e.g., hotels); in England, it is a maximum of 30 people. Additionally, specific areas, including Glasgow, North and South Lanarkshire, East and West Dunbartonshire, Renfrewshire, and East Renfrewshire have been informed of tighter restrictions and include not meeting other households indoors. Police Scotland have the power to break up both house parties and parties held within university student accommodation. The British media are reporting how the Prime Minister Boris Johnson is not ruling out another national lockdown, based on the request of the Chief Medical Officer who wants the UK government to impose a two-week national lockdown [183]. The Mayor of London Sadiq Khan has already announced celebrations for New Year’s Eve cancelled [184].

With these ever-changing directives during this pandemic, frameworks such as the ‘Smart Age-friendly Ecosystem’ [30] and the newly proposed ‘Concept of Age-friendly Smart Ecologies (CASE)’ framework illustrates how a myriad of actors across various levels of society can adapt respective ecosystems accordingly. This can be from the physical space of the individual home to the community space, or a specific organization/community hub (e.g., place of worship, educational institute, or business). Taking a locality approach may afford all respective and key actors the opportunity to share knowledge, evidence, and use and implement the voice of the residents/users with those who are playing key roles (e.g., councils, teachers, construction companies, and health practitioners).

Earlier on in this paper, we provided an array of frameworks that should be considered for future work, which could consider a combination of frameworks and universal design measures [173] as a means of assessing the framework presented here and by the respective work by Marston and van Hoof [30], Shin [70], Shin and Park [124] to afford this work to be taken to the next phase(s), while advancing the discussion of respective ecosystems and age-friendly narratives forward.

This may include a mixed-method approach comprising of qualitative and quantitative data collections—the former whereby various actors are interviewed to understand the needs, expectations, requirements, and impacts of the different hubs and spheres that make up the ‘Concept of Age-friendly Smart Ecologies (CASE)’ framework and/or ‘Smart Age-friendly Ecosystem’ (SAfE) [30] frameworks, while ensuring there is a co-production approach with stakeholders, business(es), users/residents, policy makers, technologists, gerontechnologists, social scientists, urban planners, geographers, economists, health practitioners, and gerontologists. Incorporating such a broad breadth of actors who represent different areas within the various levels of this framework has the potential to provide a rich amount of qualitative data.

The latter—quantitative data can provide similar affordances to these actors, as demonstrated by Dikken and colleagues [185], who recently published their validated age-friendly survey, the ’Age Friendly Cities and Communities Questionnaire (AFCCQ)’. This survey comprises of 23 items in the domains of: 1. Housing, 2. Social participation, 3. Respect and Social inclusion, 4. Communication and information, 5. Community support and health services, 6. Outdoor spaces and buildings, 7. Transportation and 8. Financial situation. The development and validation of this age-friendly survey has been tested on older adults and their experiences relating to the eight domains of the WHO age friendly cities model, with one extra domain—financial situation. It is anticipated and expected that the AFCCQ survey will help practitioners and researchers to understand and capture the level of age-friendly elements within a community in a quantified manner. Furthermore, there is the likelihood that this newly developed and validated tool will assist many interested researchers who are keen to understand the impact and potential benefits of age-friendly directives, policies, and social programs in the Western world.

The proposed ‘Concept of Age-friendly Smart Ecologies (CASE)’ framework is a working framework and one that can be adapted as technologies, developments, and society evolves and changes in the future. Future work should consult residents and users from various regions and communities, including areas that are both wealthy and deprived.

Torku and colleagues [132] note how both the WHO and the European Union (EU) have spearheaded the narratives of age-friendly cities and smart cities concepts and purport how these two “concepts are perceived as separate concepts” (p. 4). Yet, implementing and executing a co-design and co-producing approach to future research in this domain with actors, residents, and users from diverse populations not only produces a rich set of data, but also provides greater insight into the challenges, barriers, and enablers that various populations are faced with on a day-to-day level. Moreover, by taking a co-production approach in conjunction with the principles of universal design from both a philosophical and practical standpoint, there is the opportunity to centralize and knot together these two concepts (age-friendly cities and smart cities) [132]. We believe that the *CASE* framework can facilitate this nexus and afford future evaluations to be conducted using the framework presented here.

Understanding the challenges, barriers, and enablers which citizens are currently facing and may face in the future can offer research teams, policy makers, technologists, planners, and developers the opportunity to tailor areas specifically needed by residents in a town, city, or region [186].

To ensure that the voice of residents and users is heard, research and development (R&D) teams should aim to build strong and trustworthy relationships with communities that may or may not be hard to reach, and who may be skeptical of the intentions of policy makers, and R&D teams in delivering their needs, requirements, and expectations appropriately.

We would like to open this discussion further with various actors, policy-makers, researchers, and developers in a bid to move the debate of age-friendly forward to ensure a lasting legacy can be achieved and also adapted in an agile way.
